# Developmentally Inspired Bioprinting of Nascent Multicellular Human Heart Tissue Through In Situ Differentiation and Morphogenesis of iPSCs

**DOI:** 10.1002/advs.202522241

**Published:** 2026-05-20

**Authors:** Ankita Pramanick, Juhi Chakraborty, Orlaith Kennedy, Hey Wei Wong, Sogol Kianersi, Daniel Kelly, Vasileios Sergis, Abhay Pandit, Andrew C. Daly

**Affiliations:** ^1^ CÚRAM Research Ireland Centre For Medical Devices University of Galway Galway Ireland; ^2^ Biomedical Engineering School of Engineering College of Science and Engineering University of Galway Galway Ireland

**Keywords:** embedded bioprinting, granular hydrogels, In situ cardiac differentiation, iPSCs, shape‐morphing

## Abstract

Current approaches to heart tissue bioprinting typically rely on using human induced pluripotent stem cell (iPSC)‐derived cardiomyocytes that are pre‐differentiated in 2D culture. This differs fundamentally from embryonic heart development, where mesodermal progenitors differentiate into cardiomyocytes within 3D, matrix‐rich, and shape‐morphing microenvironments. Here, we introduce a developmentally inspired approach that enables in situ mesodermal and cardiac differentiation of iPSCs within bioprinted, shape‐morphing pluripotent tissues. Using embedded bioprinting, Matrigel bioinks with high‐density iPSC suspensions were deposited into granular support hydrogels to generate pluripotent tissue constructs with defined architectures. These constructs exhibited shape‐morphing behavior, tunable by modulating the support bath viscoelasticity. Support bath mechanics also regulated iPSC fate, with softer formulations reducing spontaneous differentiation. Building on this, mesodermal and cardiac differentiation were directly driven within the morphing constructs via temporal WNT pathway modulation, resulting in multicellular cardiac tissues in which cardiomyocytes and fibroblasts co‐emerge from a common progenitor pool. These nascent heart tissues exhibited a developmental phenotype, with immunofluorescence and gene expression profiling revealing cardiac progenitors alongside maturing cardiomyocytes. Together, these findings highlight the potential for an alternative developmental biofabrication paradigm focused on printing pluripotent organ rudiments that recapitulate early aspects of embryonic development via programmed in situ lineage specification and shape‐morphing.

## Graphical abstract

A developmentally inspired bioprinting approach enables the fabrication of pluripotent tissues that undergo shape‐morphing and in situ cardiac lineage specification. This method employs embedded bioprinting to deposit iPSCs within soft granular hydrogels to create pluripotent tissue constructs that undergo cell‐mediated shape morphogenesis. Temporal modulation of WNT signalling directs mesodermal induction and cardiogenesis within the morphing constructs, generating nascent human heart tissues in which cardiomyocytes and fibroblasts co‐emerge from a common progenitor pool.

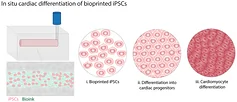

## 1 Introduction

Bioprinting has emerged as a powerful approach for fabricating human cardiac tissues by combining induced pluripotent stem cells (iPSCs), bioinks, and additive manufacturing [1, 2, 3, 4]. Most cardiac bioprinting strategies load bioinks with iPSC‐derived cardiomyocytes (iPSC‐CMs) that have been pre‐differentiated in 2D culture, before printing them into 3D constructs with defined geometries. This paradigm has yielded promising results, producing contractile cardiac tissue constructs that exhibit spontaneous and synchronous contractions for several months [2]. Additionally, co‐culturing iPSC‐CMs with supporting fibroblasts and endothelial cells can enhance tissue organization and functional maturation [3, 5]. However, pre‐differentiation strategies differ significantly from the development of the embryonic heart. For example, iPSC‐CMs are generated in 2D microenvironments, then dissociated and re‐embedded into bioinks, steps that disrupt their native cell‐matrix and cell‐cell interactions. This approach bypasses the natural developmental trajectory from pluripotency to mesodermal progenitors to cardiomyocytes, which occurs within a 3D, matrix‐rich, and mechanically evolving microenvironment. Furthermore, supporting cells, such as fibroblasts and endothelial cells, are typically generated using separate differentiation protocols, limiting the intercellular signalling that accompanies developmental co‐emergence.

Parallel progress in cardiac organoid engineering has shown that ‘in situ’ differentiation of iPSCs can generate multicellular tissues that recapitulate key features of early heart development [6, 7]. In these systems, iPSCs differentiate into mesodermal progenitors, followed by cardiac lineage specification, leading to the co‐emergence of multiple cardiac cell types, including cardiomyocytes, endothelial cells, and fibroblasts [6, 7, 8]. Importantly, these self‐organizing organoids or cardioids also exhibit spatial patterning of myocardial, endocardial, and epicardial cells and can form cavity‐containing structures reminiscent of nascent heart chambers [6]. Employing such biomimetic ‘in situ’ differentiation approaches for cardiac bioprinting holds great promise, as printing affords greater geometric control and scalability compared to organoid systems. One study has demonstrated the successful in situ differentiation of bioprinted iPSCs into iPSC‐CMs within GelMA/collagen‐based bioinks to produce contractile heart tissue [9]. However, the core areas of these constructs became acellular by the end of the culture period, potentially due to the use of a covalently crosslinked bioink that will disrupt the cell‐cell interactions essential for iPSC survival and self‐organization in 3D matrices.

Developing bioinks that support iPSC viability and self‐organization during the early stages of culture is a critical consideration for in situ differentiation strategies. For example, it is well established that iPSCs and embryonic stem cells require cell‐cell contact and aggregation for survival [10, 11, 12, 13]. The bioink must be capable of promoting intercellular interactions following encapsulation, and viscoelastic matrices have been shown to enhance iPSC viability, proliferation, and morphogenesis [14, 15]. Beyond cell‐scale behaviors, tissue‐scale morphogenesis is also a critical consideration. During embryogenesis, cardiac differentiation coincides with morphogenetic processes, such as heart tube formation and looping [16, 17, 18]. Inspired by this, our prior work has shown that cell‐mediated shape‐morphing can enhance the structural and functional properties of bioprinted heart tissues [5]. In these studies, the contractile forces generated by fibroblasts induced programmable shape‐morphing in collagen‐based bioinks, with the resulting internal stresses promoting cardiomyocyte alignment and sarcomere maturation. However, because these constructs were bioprinted using pre‐differentiated iPSC‐CMs and adult cardiac fibroblasts, morphogenetic shape changes were decoupled from the early differentiation transitions.

Here, we present a developmentally inspired bioprinting strategy that combines shape‐morphing with in situ cardiac lineage specification. Using embedded bioprinting, we deposited Matrigel bioinks containing high‐density suspensions of iPSCs (150 million cells mL^−1^) into soft granular support hydrogels that preserved the print geometry while permitting cell‐driven morphogenesis. We first sought to identify support bath formulations that maintain iPSC pluripotency while minimizing spontaneous differentiation. Building on this, we then aimed to direct mesodermal differentiation and cardiogenesis within these shape‐morphing constructs by temporally modulating the WNT pathway. We hypothesized that synchronizing differentiation with morphogenesis would produce nascent cardiac tissue capable of endogenous structural and functional maturation.

## 2 Main

### 2.1 Bioprinting iPSCs in Granular Support Hydrogels

**FIGURE 1:**
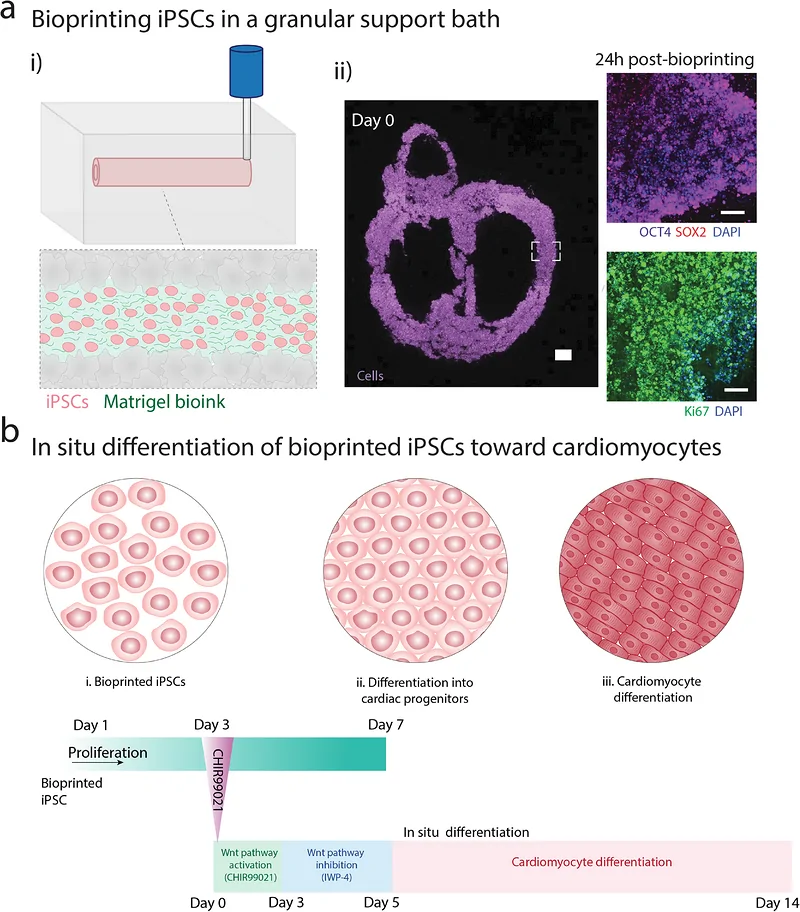
Embedded bioprinting of iPSCs in a granular bath supporting proliferation and in situ differentiation: (a) (i) Schematic representation of iPSC bioprinting in a granular support bath. (ii) Confocal image of the chambered heart cross‐section bioprinted in an agarose support bath (left, scale 1000 µm) and immunofluorescence staining of pluripotency (OCT4, SOX2) and proliferation (Ki67) markers after bioprinting (scale 100 µm). (b) Schematic illustration of bioprinted iPSCs undergoing proliferation within the bioink prior to differentiation into cardiomyocytes via WNT pathway modulation using the modified GiWi protocol [19].

First, we established a method for bioprinting 3D constructs composed of iPSCs. To achieve this, iPSCs were suspended at a high density in a Matrigel‐based bioink and extruded into agarose granular support baths using an embedded bioprinting technique (Figure 1a (i, and ii)). The Matrigel bioink was maintained at 6–8°C in a non‐gelled state to enable extrusion (Figure S1). The shear‐thinning and self‐healing properties of the granular support hydrogel facilitated precise deposition of the low‐viscosity bioink, allowing for the fabrication of complex 3D architectures (Figure 1a (i, ii)). The agarose bath is thermally stable at 37°C and non‐degradable, thereby providing structural support to the soft bioink filaments during the early stages of culture. Confocal imaging 24 h post‐printing confirmed that iPSCs within the printed constructs expressed the pluripotency markers OCT4 and SOX2, as well as the proliferation marker Ki67, indicating the maintenance of stemness and active cell division (Figure 1a (ii)). After demonstrating that undifferentiated iPSCs could be bioprinted into 3D constructs within granular support hydrogels while retaining pluripotency, the next objective was to investigate whether these cells could undergo in situ mesodermal induction and differentiate into cardiomyocytes directly within the bioink (Figure 1b). To optimize the efficiency of this in situ differentiation process, we next examined how the composition of both the bioink and the granular support hydrogel could impact iPSC viability and differentiation post‐printing.

**FIGURE 2:**
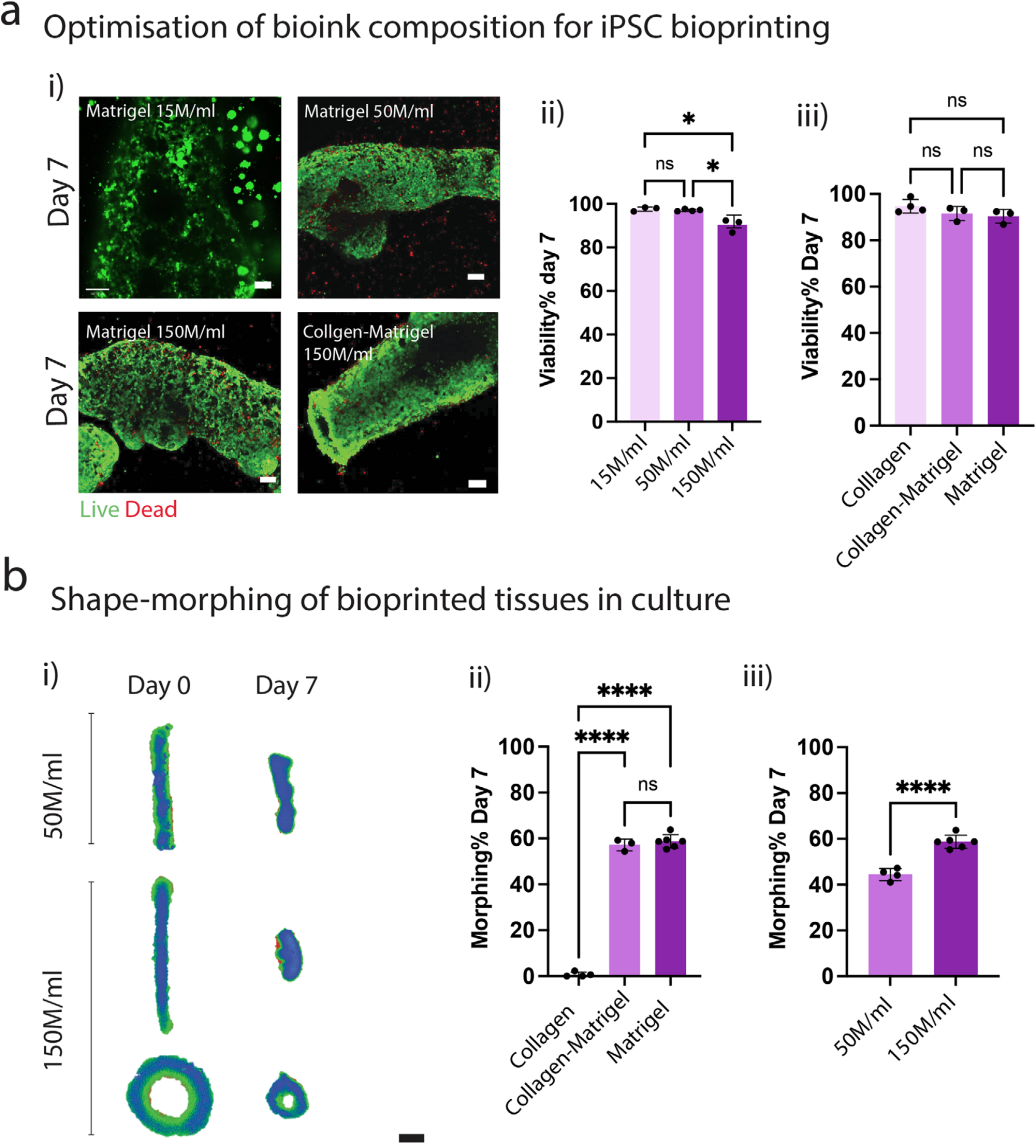
Evaluation of cell viability and tissue shape‐morphing of bioprinted constructs: (a) (i) Confocal images indicating the viability within the bioprinted iPSC tissues (scale 100 µm) and (ii, iii) quantitative analysis demonstrating the percentage of live cell area as a function of cell density (15, 50, 150 million cells/mL) and ink composition (collagen, collagen + Matrigel, Matrigel), respectively, on day 7. (b) Bioprinted constructs exhibited tissue shape‐morphing during the culture period, as evidenced by (i) brightfield images showing the change in tissue dimension over 7 days (scale 1 mm). Quantitative analysis of shape‐morphing during in situ differentiation through measurement of ring diameter or beam length over time. See the methodology section ‘microscopy analysis of bioprinted constructs’ for the formula used to calculate the shape‐morphing %. Shape‐morphing is plotted as a function of (ii) bioink composition and (iii) cell density (n = 3 to 6 biological replicates, unpaired t‐test for two groups and one‐way ANOVA with Tukey's multiple comparison test for more than two groups, where ns denotes not significant, ^*^ denotes *p* < 0.05, and ^****^ denotes *p* < 0.0001).

Bioprinting iPSCs as single‐cell suspensions presents challenges due to their sensitivity and dependence on cell‐cell interactions for survival [10]. These interactions are essential for maintaining pluripotency and preventing spontaneous differentiation. In addition, external stresses, such as shear forces during extrusion or unfavorable bioink properties, can compromise viability and pluripotency. To mitigate these effects, high‐density iPSC suspensions (15‐150 million cells mL^−1^) were used for bioprinting to enhance cell‐cell interactions. Constructs bioprinted with the highest cell density (150 million cells mL^−1^) maintained approximately 90% viability for up to 7 days of culture (Figure 2a (i, ii) and Figure S2a). Although lower cell densities (15 and 50 million cells mL^−1^) exhibited slightly higher viability (>95%), these constructs were mechanically weaker and prone to structural failure during culture (Figure S2b (i, ii, iii)). In contrast, constructs bioprinted at 150 million cells mL^−1^ displayed consistent structural integrity, likely due to enhanced cell‐cell adhesion. Confocal imaging on day 1 confirmed that the iPSCs were densely packed within the bioprinted constructs (Figure 1a (ii)). Ki67 staining also indicated active iPSC proliferation (Figure 1a, ii), which likely further enhanced cell‐cell adhesion and construct stability.

The impact of bioink composition on iPSC behavior was assessed, focusing on Matrigel and collagen because of their known capacity to support cell‐extracellular matrix (ECM) interactions. Matrigel is a well‐established matrix for iPSC culture [20, 21], and collagen hydrogels have also been used for 3D culture of iPSCs [22, 23]. Three formulations were examined: collagen type I alone (0.96 mg mL^−1^), Matrigel alone (3.2–4.4 mg mL^−1^), and a mixed bioink containing both collagen (0.6 mg mL^−1^) and Matrigel (2–2.75 mg mL^−1^). Although we did not quantify stiffness directly, the printed constructs gradually stabilized or stiffened during culture within the support hydrogel, likely due to the formation of cell‐cell adhesions and/or nascent ECM production. Our previous work has demonstrated that bioprinted constructs stiffen during culture as they undergo cell‐mediated shape‐morphing [5]. The printed constructs were cultured within the support hydrogels for 3 days until they established sufficient structural integrity to be removed via the gradual dilution of the support bath with culture media. All bioink formulations supported high cell viability (≥ 90%), with no significant differences observed between groups (Figure 2a (iii)). However, live/dead staining revealed that iPSC morphology varied with bioink composition. For example, iPSCs encapsulated in collagen‐only bioinks remained largely dispersed as single cells (Figure S2a (i)), whereas both Matrigel‐only and collagen‐Matrigel bioinks promoted cell aggregation and intercellular connectivity (Figure S2a (i)). This enhanced cell aggregation is likely due to Matrigel containing proteins such as laminin, collagen IV, and entactin, which support cell adhesion and survival through integrin signalling pathways [20, 21].

Cell viability was further compared between iPSCs bioprinted as single‐cell suspensions and those printed as pre‐formed embryoid bodies (EBs) within the Matrigel bioink. The EB culture format is commonly used for iPSC culture as it promotes cell‐cell contact and, in the context of extrusion bioprinting, may protect iPSCs from shear stress during extrusion. Despite these potential advantages, both bioprinting approaches using single‐cell suspensions or EBs achieved similar high cell viabilities of approximately 90% (Figure S2a (i, ii)). By day 7, constructs printed from single‐cell suspensions exhibited robust cell‐cell connectivity (Figure 2a (i)), indicating that the iPSCs had self‐assembled into geometrically defined 3D structures with dense cellular packing comparable to that of EBs. These results demonstrate that high‐density iPSC suspensions in Matrigel bioinks can rapidly establish intercellular connectivity following printing, generating cell‐dense, structurally stable, pluripotent tissue constructs. Based on these results, the Matrigel bioink with a cell density of 150 million mL^−1^ was selected for further studies, unless otherwise stated.

### 2.2 Shape‐Morphing of Bioprinted iPSC Constructs in Granular Support Hydrogels

Granular support hydrogels provide a soft viscoelastic environment that can accommodate the shape‐morphing of bioprinted constructs. For example, our previous work has demonstrated that fibroblast‐generated contraction forces can drive the shape‐morphing of collagen hydrogels in granular supports [5]. Similarly, in the present study, bioprinted iPSC‐containing constructs underwent significant shrinkage and shape‐morphing during culture (Figure 2b). The constructs were printed in a soft support bath formulation (storage modulus ≈ 105 Pa), which provided structural support during the early stages of culture while also enabling structural shape transformations. The support bath was gradually diluted and removed by day 3, once the constructs had developed sufficient structural integrity. The magnitude of shape‐morphing was influenced by both the bioink composition and cell density (Figure 2b). For example, constructs bioprinted with Matrigel or collagen‐Matrigel bioinks underwent greater shape‐morphing than those printed with collagen alone, which remained largely static (Figure 2b (ii)). Additionally, increasing the iPSC density from 50 to 150 million cells mL^−1^ significantly enhanced shape‐morphing in Matrigel‐based constructs (Figure 2b (iii)). In 2D culture, iPSC colonies have been shown to maintain their morphology and compactness through contractile actin fences and focal adhesions [24]. These contractile actin fences exert Rho‐ROCK‐myosin mechanical stresses to maintain colony morphology and compaction. The shape‐morphing observed in our bioprinted constructs may be driven by similar mechanisms, whereby iPSCs contract the Matrigel matrix to form intercellular adhesions and densely packed cellular microarchitectures. The constructs bioprinted with both beam‐ and ring‐shaped geometries displayed comparable degrees of shape‐morphogenesis (Figure S2c (i, ii)). In both ring‐shaped and beam constructs, morphing increased significantly over time from ∼30% to 38% on day 3 to ∼55%–57% by day 7 (Figure S2c (iii)). It should be noted that some loss of the outer cell and bioink layers occurred within the initial 72 h, but overall, the constructs maintained the desired bioprinted structure (e.g. beam or ring).

**FIGURE 3:**
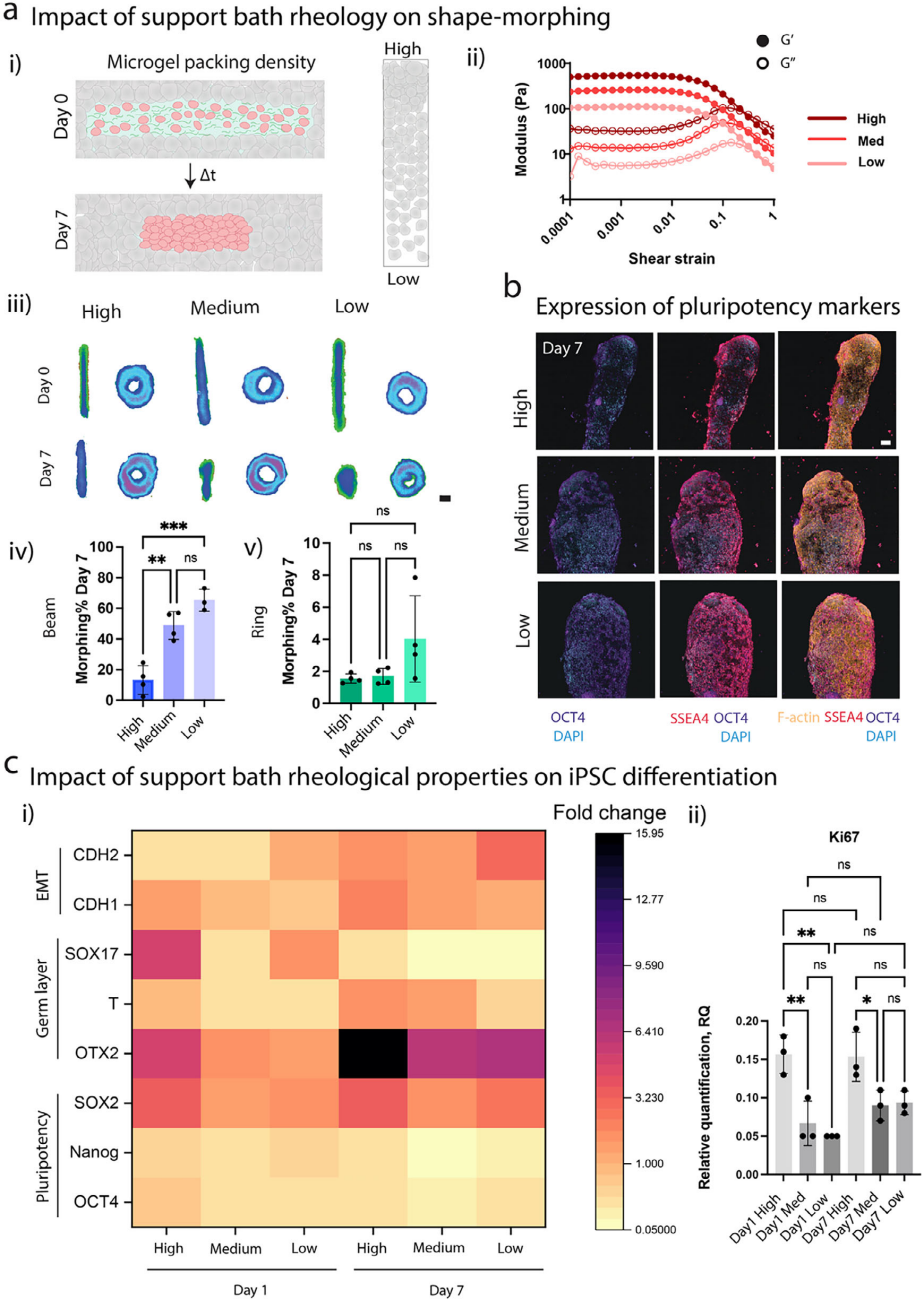
Influence of support bath packing density on tissue shape‐morphing: (a) (i) Schematic representation of tissue shrinkage in the granular support bath varying the microgel packing density from high (100%) to low (60%). (ii) Amplitude sweep indicated the transition of elastic to fluid‐like behavior of all formulations, modulating the microgel packing density. (iii) Brightfield images (scale bar 1 mm) and quantitative analysis showed the influence of varying packing density on shape‐morphing in seven‐day culture in (iv) beam construct (% change in length) and (v) ring geometry (% change in diameter) (n = 3/4 biological replicates, one‐way ANOVA with Tukey's multiple comparison test, where ^**^ denotes *p* < 0.01 and ^***^ denotes *p* < 0.001). See the methodology section ‘microscopy analysis of bioprinted constructs’ for the formula used to calculate the shape‐morphing %. (b) Immunofluorescence staining indicated the expression of pluripotency markers (OCT4, SSEA4) in support bath with varying packing densities (scale 100 µm) on day 7. (c) (i) Heatmap demonstrating pluripotency, germ layer, EMT, and proliferation‐related gene expression analysis of bioprinted iPSC constructs in high, medium, and low packing densities, with GAPDH as the housekeeping gene and iPSCs cultured in 2D conditions as the control sample (n = 3 biological replicates). One‐way ANOVA with Tukey's multiple comparison test was performed for individual genes (Figure S3). (ii) The plot indicates the fold‐change in the proliferation marker Ki67 (relative to iPSCs cultured in 2D conditions, n = 3 biological replicates). One‐way ANOVA with Tukey's multiple comparison test was performed for individual genes (Figure S3), and the heatmap is a consolidated version of this data.

To assess how the surrounding mechanical environment affected shape‐morphing, constructs were bioprinted and cultured in granular support baths with varying microgel packing densities: high (100%), medium (80%), and low (60%). All formulations exhibited yield stress behavior, transitioning from elastic to fluid‐like states at shear strains of approximately 0.1% (Figure 3a (i, ii)). As the packing density decreased from 100% to 60%, the storage modulus decreased from approximately 501 to 105 Pa (Figure 3a (i, ii)). The constructs were cultured in the support bath for 7 days (without dilution or removal of the support). As anticipated, the extent of shape‐morphing was influenced by the relative elasticity of the support bath. Beam constructs cultured in the stiffest bath (100% packing) underwent the least shape‐morphing (13%), whereas constructs cultured in the softest bath (60% packing) underwent significantly greater shape‐morphing (65%) (Figure 3a (iii, iv)). This indicates that the elasticity of the granular support bath can be tuned to modulate the extent of shape‐morphogenesis. Interestingly, the ring‐shaped constructs exhibited minimal shape‐morphing across all packing densities (∼1.5%–4% change in diameter) (Figure 3a (iii, v)). These lower levels of shape‐morphing observed in the bioprinted rings are likely due to the geometric constraints imposed by the closed‐loop architecture, where support gel is retained within the ring's core, providing mechanical resistance to morphing. In contrast, beam constructs likely experience less structural confinement and can contract more freely along their longitudinal axis.

### 2.3 Impact of Support Bath Mechanics on iPSC Fate in Shape‐Morphing Constructs

Next, we examined how the support bath mechanics influenced iPSC pluripotency and differentiation within the shape‐morphing constructs. Immunofluorescence staining confirmed the expression of pluripotency markers (OCT4 and SSEA4) up to day 7 (Figure 3b). To further assess iPSC fate, temporal gene expression analysis was performed on days 1 and 7 post‐printing using qRT‐PCR. The analysis included pluripotency‐associated genes (OCT4, Nanog, and SOX2), germ layer‐specific genes (OTX2 for ectoderm, Brachyury (T) for mesoderm, and SOX17 for endoderm), and the proliferation marker (Ki67). All results were normalized to those of iPSCs cultured in 2D conditions. The expression of epithelial‐to‐mesenchymal transition (EMT)‐related genes was also evaluated. E‐cadherin (CDH1) is essential for iPSC self‐renewal, and its expression is associated with the undifferentiated iPSC state [25]. Conversely, N‐cadherin (CDH2) expression is associated with lineage differentiation [25, 26, 27]. The expression of OCT4 and Nanog decreased over 7 days of culture in the 60% packing density support (21% and 70% reduction, respectively) (Figure 3c (i) and Figure S3a,b). OCT4 and Nanog expression also decreased in the stiffer 100% packing density support (50% and 54% reduction, respectively) (Figure 3c (i) and Figure S3a, b). SOX2 expression was stable across all formulations. At day 7, the expression of the three pluripotency markers did not differ across the different stiffness supports (Figure 3c (i) and Figure S3a, b). Notably, the expression of OTX2 (ectoderm marker) increased over 7 days across all stiffness formulations, with the highest expression (fold‐change ∼16) observed in the stiffest bath (Figure 3c (i) and Figure S3a, b). This suggests that the increased physical constraint provided by a stiffer support bath can impact iPSC differentiation within the constructs during morphing. Ki67 expression also increased in the stiffer support bath, indicating enhanced proliferation (Figure 3c (ii)). Slight increases in CDH1 expression (E‐cadherin, associated with an undifferentiated state) were also observed in the stiffer baths (Figure 3c (i) and Figure S3a, b). Finally, no significant changes in SOX17 (endoderm) or T‐Brachyury (mesoderm) expression were observed between the conditions on day 7 (Figure 3c (i) and Figure S3a, b).

**FIGURE 4:**
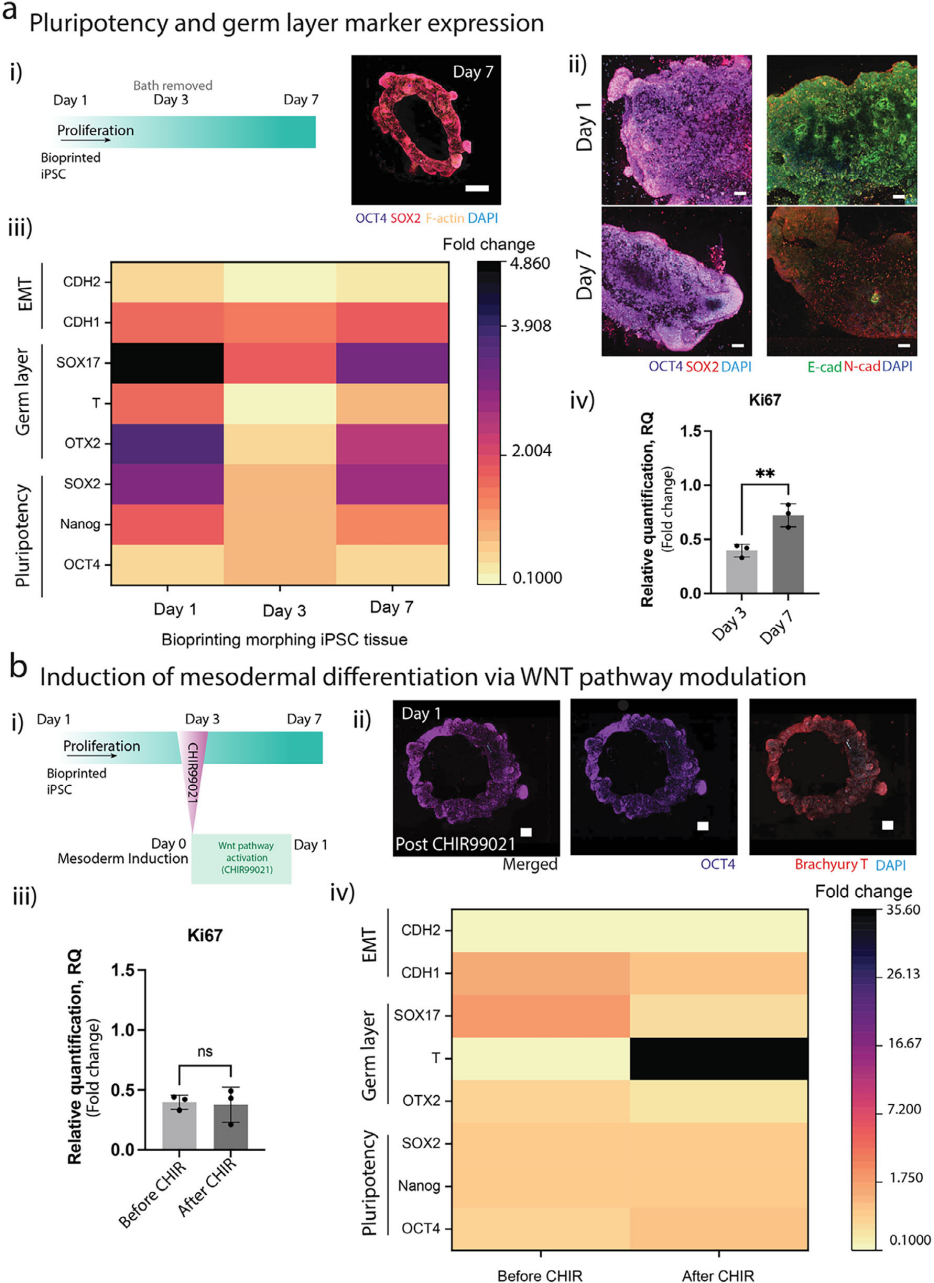
Expression of pluripotency, germ layer, and EMT‐associated genes within the bioprinted iPSCs morphing tissue in culture and upon mesodermal induction: (a) (i) Schematic representation of the time points day 1, 3, and 7 to evaluate temporal change in expression of pluripotency (OCT4, Nanog, and SOX2), germ layer (OTX2, T, SOX17), and epithelial‐to‐mesenchymal transition (CDH1 and CDH2) via qRT‐PCR. (Right) Confocal image of a bioprinted ring on day 7 (scale 500 µm). (ii) Immunofluorescence images of bioprinted tissue highlighted the expression of pluripotency markers (OCT4 and SOX2) and EMT markers (E‐cadherin and N‐cadherin) on days 1 and 7 (scale 100 µm). (iii) The heat map demonstrates the temporal change in expression during the culture period with GAPDH as the housekeeping gene and iPSCs cultured in 2D conditions as the control sample (n = 6 biological replicates). (iv) Quantitative analysis of the proliferation marker Ki67 as fold‐change value on days 3 and 7 relative to day 1 bioprinted tissue (n = 3 biological replicates, unpaired *t*‐test, where ^**^ denotes *p*< 0.01). (b) (i) Schematic flow representing the time points selected to evaluate pluripotency, germ layer, and epithelial‐mesenchymal transition (EMT) markers before and after WNT activation using CHIR99021. (ii) Confocal images of the bioprinted ring upon mesoderm induction on differentiation day 1 showed immunostaining for OCT4 and the mesoderm marker Brachyury (T) (scale 200 µm). (iii) The plot indicates the fold‐change in the proliferative marker Ki67 (relative to day 1 samples) before and after mesoderm induction on differentiation day 0 (or day 3 post‐bioprinting) and day 1 (post‐CHIR99021) relative to day 1 post‐bioprinted iPSCs (n = 3 biological replicates, unpaired *t*‐test, where ns denotes not significant). (iv) The expression of genes associated with pluripotency, germ layer, and EMT was evaluated and represented as a heat map, with GAPDH as the housekeeping gene and 2D iPSCs as the control sample (n = 6 biological replicates). Unpaired t‐test and one‐way ANOVA with Tukey's multiple comparison test were performed for individual genes (Figures S4 and S5). Heatmaps are a consolidated version of this data.

Next, we investigated whether removing the support bath on day 3 of culture affected iPSC differentiation. As our goal was to subsequently induce cardiac differentiation by modulating the WNT pathway, we sought a bath formulation that maintained pluripotency while minimizing spontaneous differentiation. The softer support bath formulation (60% packing density) was selected for these studies, as it enhanced shape‐morphing while reducing the expression of iPSC differentiation markers (Figure 3a,c). The rationale for subsequently removing the soft support bath in the later stages of shape‐morphing was two‐fold. First, removal eliminates potential diffusion barriers to the delivery of the small molecules required for subsequent cardiac specification. Second, given that softer baths were associated with reduced spontaneous differentiation, removing the support entirely was expected to result in a purer population of pluripotent stem cells prior to the induction of cardiac differentiation. Temporal gene expression analysis was performed on shape‐morphing constructs on days 1, 3, and 7. Notably, no significant changes in pluripotency marker expression were observed over 7 days (Figure 4a (iii) and Figure S4). Additionally, immunofluorescence staining for OCT4 and SOX2 was observed on day 7 (Figure 4a (ii) and Figure S4b). The expression of germ‐layer markers also remained unchanged during the culture period (Figure 4a (iii) and Figure S4b). These results suggest that the iPSC population remained pluripotent when cultured in softer support hydrogels during the early stages of the shape‐morphing process. It should be noted that E‐cadherin staining (associated with undifferentiated cells) appeared to decrease over 7 days of culture (Figure 4a (ii)); however, no significant changes in CDH1 or CDH2 expression were observed by day 7 (Figure 4a (iii) and Figure S4b). Finally, between days 3 and 7, Ki67 expression increased by 82%, suggesting active iPSC proliferation within the constructs (Figure 4a (iv)).

Collectively, these results demonstrate that the stiffness of the support bath can modulate the fate of iPSCs within shape‐morphing constructs. The stiffest bath, which restricted morphing, induced a significant upregulation of the ectodermal marker OTX2. Our previous study demonstrated that stiffer granular supports increase stress and strain within bioprinted shape‐morphing constructs [5]. Mechanical cues have been shown to impact iPSC differentiation [28, 29], which likely contributed to the elevated expression of OTX2 in stiffer support baths. In contrast, softer support hydrogels supported greater morphing while simultaneously suppressing spontaneous lineage commitment. Furthermore, when the softer support bath was removed after three days, pluripotency was maintained. It should be noted that this experiment was only performed with an agarose support bath. Agarose is a relatively bioinert hydrogel that lacks native cell‐binding domains, allowing bioprinted constructs to undergo shape‐morphing freely within the support without any cell‐adhesive effects that could prevent morphing. The observed differences in iPSC fate as a function of packing density are therefore likely primarily driven by the physical stiffness of the support material; however, testing additional support materials with tunable mechanical properties could help decouple any potential material‐specific effects from stiffness.

Overall, these results indicate that the embedded bioprinting of iPSCs within softer granular support baths enables the fabrication of cell‐dense, pluripotent 3D constructs with defined architectures. The maintenance of pluripotency throughout the culture period highlights the potential for subsequent directed mesodermal and cardiac lineage differentiation through the biochemical modulation of the WNT pathway during shape‐morphogenesis. Accordingly, the next phase of the study focused on developing mesodermal and cardiac differentiation protocols for bioprinted shape‐morphing constructs.

### 2.4 Guided In Situ Mesodermal and Cardiac Lineage Differentiation Within Bioprinted Constructs

Building on the findings that pluripotency was preserved during shape‐morphing for at least 3 days in softer support bath formulations, we next explored in situ mesodermal and cardiac differentiation by modulating WNT signalling. On day 3 post‐bioprinting, WNT signalling was activated using the GSK3 inhibitor CHIR99021 to induce mesodermal specification (Figure 4b). To evaluate the effects of WNT activation, qRT‐PCR was performed before and after CHIR99021 addition (Figure s4b (iii, iv) and Figure S5). A significant upregulation (∼35 fold change) of brachyury (T), a T‐box transcription factor and mesodermal marker, was observed (Figure 4b (iv) and Figure S5). The expression levels of OTX2 (ectoderm) and SOX17 (endoderm) remained unchanged, indicating that differentiation was restricted to the mesodermal lineage only. The expression of E‐cadherin, N‐cadherin, and Ki67 also remained unchanged in response to WNT activation (Figure 4b (ii, iv) and Figure S5). These results demonstrate the successful induction of mesodermal differentiation in situ within shape‐morphing tissues, highlighting the potential of using targeted signalling modulation to recapitulate developmental‐like iPSC lineage specification in bioprinted constructs.

**FIGURE 5:**
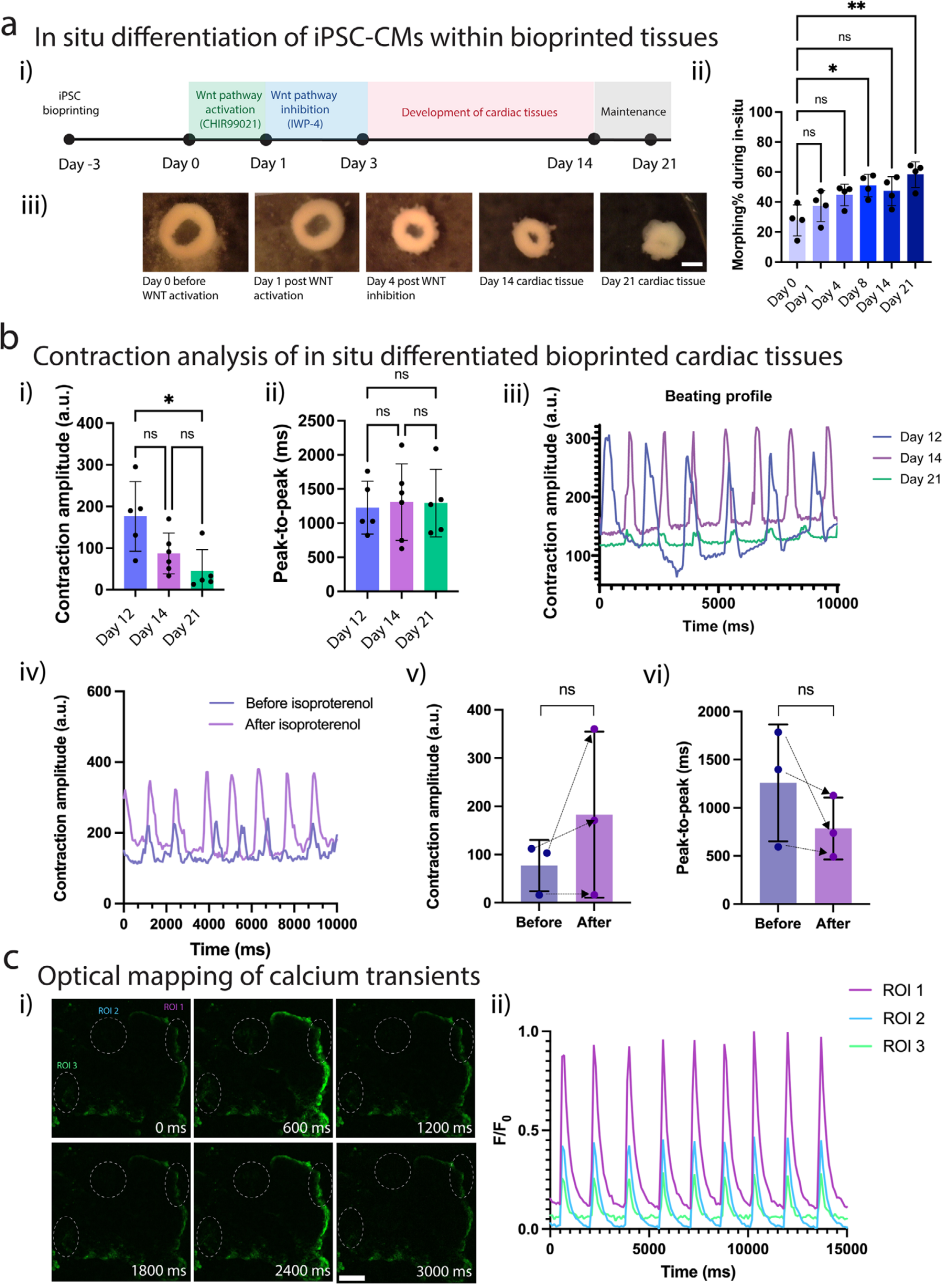
In situ differentiation of bioprinted pluripotent shape‐morphing constructs into cardiac tissue: (a) (i) Schematic overview of the modified GiWi protocol used to induce mesoderm and cardiac differentiation within bioprinted shape‐morphing tissues. (ii) Quantitative analysis of shape‐morphing during in situ differentiation through measurement of ring diameter over time. See the methodology section ‘microscopy analysis of bioprinted constructs’ for the formula used to calculate the shape‐morphing %. (n = 3 biological replicates, one‐way ANOVA with Tukey's multiple comparison test, where ns denotes not significant, and ^*^ denotes *p* < 0.05, ^**^ denotes *p* < 0.01). (iii) Brightfield images of bioprinted iPSC tissues before WNT activation and during in situ cardiac differentiation (scale 1 mm). (b) (i) Contraction amplitude, (ii) peak‐to‐peak time, (iii) contraction profile of in situ differentiated bioprinted cardiac tissue on days 12, 14, and 21 (n = 5/6 biological replicates, one‐way ANOVA with Tukey's multiple comparison test, where ns denotes not significant, and ^*^ denotes *p* < 0.05), (iv) Representative contraction profile of in situ differentiated bioprinted cardiac tissue before and 30 min after treatment with 1 µM isoproterenol on day 14, (v) Contraction amplitude and (vi) peak‐to‐peak time before and after treatment with isoproterenol on day 14 (n = 3 biological replicates, Student *t*‐Test, where ns denotes not significant). (c) (i) Optical mapping of calcium transients within in situ differentiated bioprinted cardiac tissue at day 14 over 3000 ms (Scale bar—50 µm), (ii) Calcium imaging traces depicting normalized fluorescence intensity (F/F_0_) in three different regions of interest (ROI). Calcium imaging was performed on n = 3 biological replicates.

Next, in situ cardiac differentiation was initiated by temporally modulating WNT signalling using CHIR99021 for activation, followed by IWP‐4 for inhibition (Figure 5a (i)). The resulting tissues were then cultured for up to 21 days, with spontaneous contractions beginning on approximately day 8–12, indicating cardiac differentiation (Figure 5b and Movie S1). The bioprinted constructs underwent shape‐morphing during the in situ differentiation process, with continued shrinkage of the tubes observed up to day 21 (Figure 5a (ii, iii)). To quantify the spontaneous contractions, the contraction amplitude (an image‐based estimation of contraction force) and peak‐to‐peak time (estimation of beating frequency) were analyzed using the open‐source Musclemotion software (Figure 5b (i, ii, iii)). The constructs exhibited a higher contraction amplitude on day 12, with significant reductions on day 21 (Figure 5b (i)). The peak‐to‐peak time remained constant between days 12 and 21, corresponding to an average beating frequency of ∼0.8 Hz (Figure 5b (ii)). This reduction in contraction force over time is potentially due to changes in ECM stiffness during culture. To assess cardiac maturity levels at day 14, constructs were treated with isoproterenol, a β‐adrenergic receptor agonist that produces positive chronotropic and inotropic effects (faster beating, stronger contraction) in cardiomyocytes (Figure 5b (iv–vi)). Although the results did not reach statistical significance, increases in contraction amplitude and decreases in peak‐to‐peak intervals (faster beating) were observed in 2 out of 3 constructs. Responsiveness to isoproterenol is indicative of functional β‐adrenergic signalling and maturing calcium‐handling machinery (e.g., SR calcium stores, SERCA2a activity) in cardiomyocytes. Next, we performed calcium imaging, which revealed spontaneous and rhythmic calcium transient activity across the bioprinted constructs (Figure 5c and Movie S2). The calcium transient durations (time from peak depolarization to 90% repolarization) ranged from 309 to 752 ms in the constructs (n = 3). This relatively large variability is likely because calcium imaging was performed on spontaneously beating constructs under non‐paced conditions.

**FIGURE 6:**
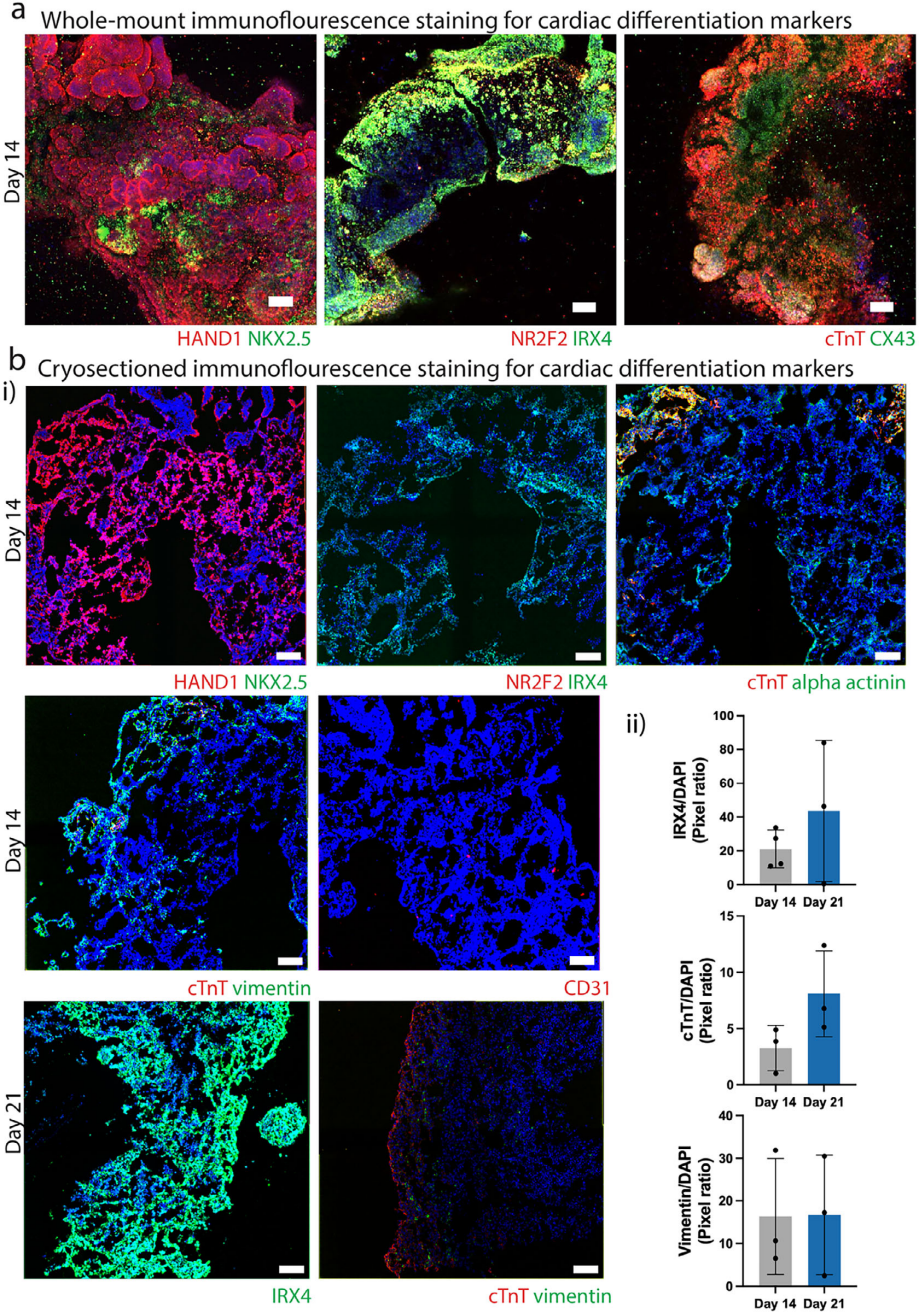
Co‐emergence of cardiomyocytes and multiple cardiac cell subtypes from iPSCs during in situ differentiation: (a) immunofluorescence images of in situ differentiated bioprinted cardiac tissues confirming the emergence of cardiomyocytes and cardiac subtypes using a panel of antibodies on day 14. The immunofluorescence analysis includes markers for cardiac progenitors (HAND1, NKX2.5), atrial cardiomyocytes (NR2F2), ventricular cardiomyocytes (IRX4), cardiomyocytes (cTnT, connexin 43), and (b) (i) immunofluorescence images of the cryosections of in situ differentiated bioprinted cardiac tissues confirming the emergence of cardiomyocytes and cardiac subtypes using a panel of antibodies on day 14 and 21. These include markers for cardiac progenitors (HAND1, NKX2.5), ventricular/atrial committed cardiac progenitors (IRX4, NR2F2), cardiomyocytes (cTnT, sarcomeric alpha‐actinin), fibroblasts (vimentin), and endothelial cells (CD31) at day 14. The expression of IRX4, cTnT, and vimentin has also been shown for day 21. (ii) Quantification of IRX4, cTnT, and vimentin expression normalized to DAPI at day 14 and 21. Data is represented as mean ± SD (n = 3, biological replicates). (Scale bars—100 µm). The specificity of these immunofluorescence markers was confirmed using negative control samples (Figure S7).

**FIGURE 7:**
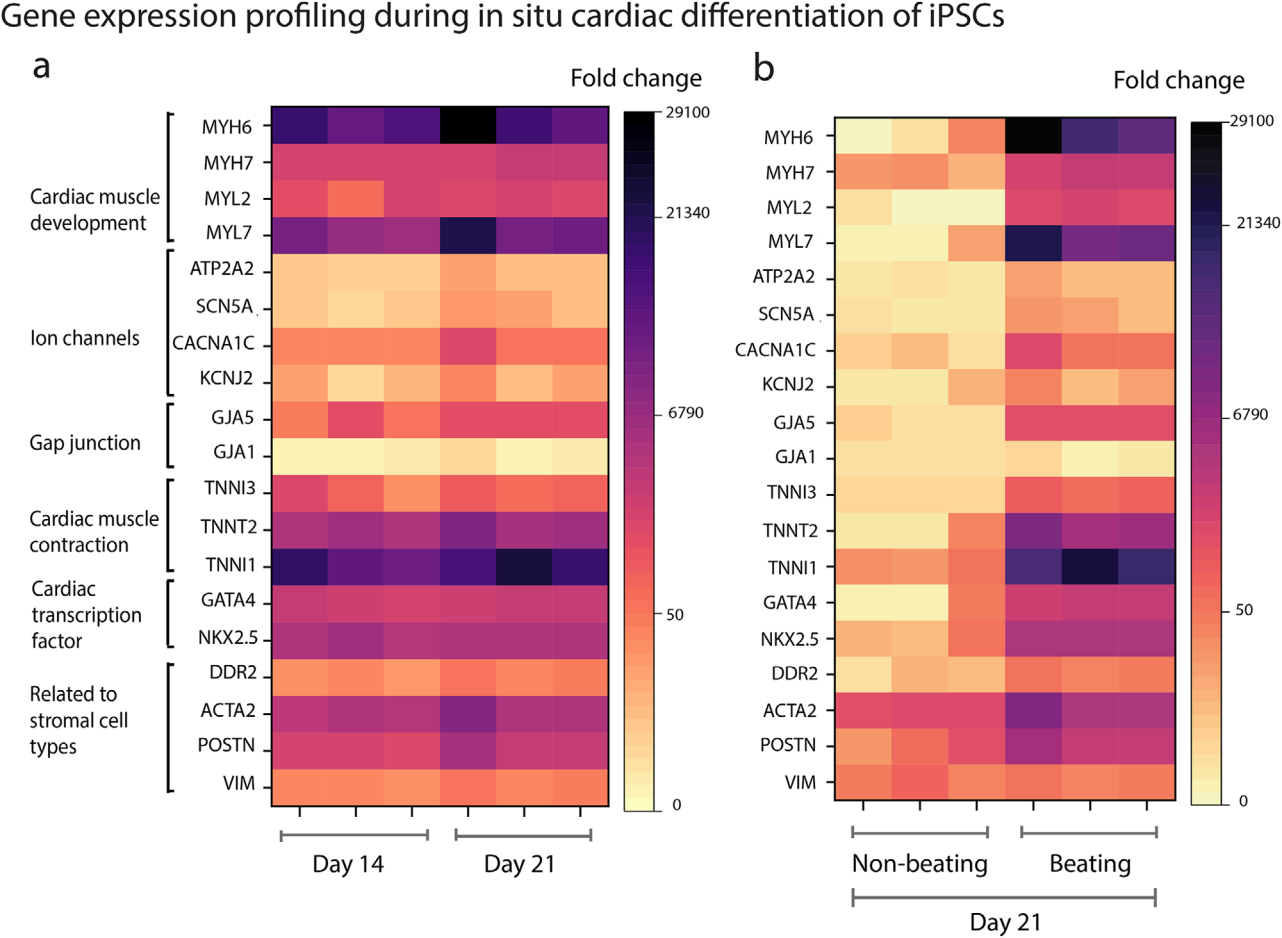
Transcriptomic profile of in situ differentiated bioprinted cardiac tissue: (a) Representation of quantitative fold‐change as a heatmap of in situ differentiated bioprinted cardiac tissue at days 14 and 21, and (b) at day 21 categorized based on their contractile properties, where non‐beating refers to the lack of spontaneous contractile forces observed post‐in situ differentiation. GAPDH was the housekeeping gene, and undifferentiated (before WNT activation) bioprinted iPSCs were the control samples (n = 3 biological replicates). A list of significantly upregulated genes is presented in tabular form in Tables S1 and S2.

Successful iPSC‐CM differentiation was confirmed by positive whole‐mount immunofluorescence staining for cardiac troponin T (cTnT) (Figure 6a and Figure S6). Additionally, we observed new tissue clusters emerging along the edges of the bioprinted constructs after WNT inhibition, which stained positively for cTnT by the end of the culture period (Figure 5a (i) and Figure S6). We also evaluated the presence of cardiomyocyte maturation markers, including connexin 43 (Cx43) and sarcomeric alpha‐actinin (Figure 6a and Figure S6). Sarcomeric alpha‐actinin staining was mostly diffuse within cTnT‐positive areas at days 14 and 21 (Figure S6), indicating the iPSC‐CMs had an immature phenotype, although some early striated sarcomeric structures had started to develop. Cx43 staining was also mostly diffuse in the cTnT‐positive areas at days 14 and 21, suggesting limited gap junction formation (Figure S6). Next, we stained for HAND1, a transcription factor marker for cardiac progenitor formation, and NKX2.5, an early marker for cardiac progenitors expressed in the developing heart tube. Positive staining for both HAND1 and NKX2.5 at day 14 confirmed that our in situ differentiation protocol initiated embryonic‐like differentiation cascades within our bioprinted tissues, with the iPSCs differentiating into cardiac progenitors followed by cardiomyocytes (Figure 6a and Figure S6). NKX2.5 expression was also significantly upregulated in our qRT‐PCR array data at day 14 (Figure 7a). We also evaluated whether the in situ differentiation protocol generated specific cardiomyocyte subtypes by co‐staining for IRX4 and NR2F2, early transcription factor markers of ventricular and atrial cardiomyocyte specification, respectively. Robust IRX4 staining and lower NR2F2 staining indicated that our in situ differentiation protocol predominantly generated ventricular‐like iPSC‐CMs (Figure 6a). Despite the higher IRX4 expression relative to NR2F2, we observed positive staining for sarcolipin (SLN) (Figure S6), a marker usually associated with atrial cardiomyocytes [30, 31]. However, this may be due to the abundance of immature progenitor cells in our tissues that potentially express low levels of SLN prior to specification [31]. We also observed positive staining for TBX18 on the surface of the constructs (Figure S6), a marker of epicardial cells [32, 33]. Finally, positive staining for collagen‐I, fibronectin, and laminin indicated that the encapsulated iPSCs were secreting nascent ECM during differentiation (Figure S6).

To evaluate the spatial distribution of cardiac differentiation within our constructs, we next performed immunofluorescence staining on cryosections (Figure 6b). Interestingly, HAND1 and IRX4 staining (indicative of ventricular committed cardiac progenitors) was distributed relatively uniformly across the cross‐sections of the tube walls at day 14, indicating spatially homogeneous cardiac induction (Figure 6b). Weak to absent NR2F2 staining on the cryosections further confirmed ventricular commitment (Figure 6b). Notably, cTnT staining was restricted to the peripheral regions of the constructs by day 14 and 21 (Figure 6b). We did observe some isolated cTnT staining within core regions by day 21 (Figure 6b (i)), but the majority of staining occurred within 100 µm of the construct surface (Figure 6b (i)). Limited alpha‐actinin‐positive sarcomeric structures were evident in these cTnT‐positive regions, indicating that the cardiomyocytes had an immature phenotype (Figure 6b). We also used the cryosectioned immunofluorescence staining to quantify IRX4, cTnT, and vimentin staining relative to DAPI (i.e., the total number of green or red pixels divided by blue pixels). The cTnT‐positive area increased from 3% at day 14% to 8% at day 21 (Figure 6b (ii)), and the vimentin‐positive area was approximately 16% at both timepoints (Figure 6b (ii)). The IRX4‐positive area was higher at both time points, 23% on day 14 and 43% on day 21 (Figure 6b (ii)). These results suggest that CHIR99021 and IWP‐4 successfully diffused into the interior to initiate cardiac progenitor specification of the iPSCs, as evidenced by uniform HAND1/IRX4 staining, but that the transition from cardiac progenitor to cardiomyocyte was restricted to more peripheral regions of the constructs. The peripheral restriction of terminal cardiomyocyte differentiation may be attributable to limitations in oxygen and/or nutrient diffusion. Oxygen diffusion in cell‐dense tissues is consumption‐limited and typically drops to hypoxic levels beyond diffusion distances of ∼100 µm in cardiac tissues [34]. During development, early cardiogenesis and cardiac progenitor specification occur in a relatively hypoxic environment [35, 36]. However, terminal cardiac differentiation requires a metabolic shift from glycolysis to oxidative phosphorylation [37]. This supports the hypothesis that insufficient oxygen and/or nutrient availability prevented cardiac progenitor‐to‐cardiomyocyte differentiation in the core regions of our constructs.

Next, we evaluated the potential co‐emergence of fibroblasts and endothelial cells with cardiomyocytes during in situ differentiation. Positive staining for DDR2 (Discoidin Domain Receptor 2), a well‐recognized marker for cardiac fibroblasts, together with vimentin (VIM), confirmed the co‐emergence of fibroblasts within the shape‐morphing constructs (Figure 6b and Figure S6). We also observed increased expression of N‐cadherin relative to E‐cadherin (Figure S6), a characteristic feature of the cadherin switch associated with cell differentiation during heart development [38, 39]. Fibroblasts were interspersed with cardiomyocytes throughout the tissue (Figure 6b and Figure S6). This co‐emerging fibroblast population likely contributed to the observed shape‐morphing during in situ iPSC differentiation. For example, our previous work demonstrated that fibroblast‐generated contraction forces can drive shape‐morphing in bioprinted heart tissues containing co‐cultures of pre‐differentiated iPSC‐CMs and cardiac fibroblasts [5]. Whole‐mount immunostaining for CD31 at day 14 demonstrated that small clusters of endothelial cells had co‐differentiated with cardiomyocytes on the surface of the constructs (Figure S6). However, cryosection staining demonstrated no CD31 expression in the core regions, suggesting limited overall endothelial cell differentiation (Figure 6b). Based on these findings, we conclude that endothelial cell co‐emergence was minimal, and our in situ differentiation protocol resulted in the co‐emergence of cardiomyocytes and fibroblasts.

To assess transcriptional changes associated with cardiac differentiation and maturation, qRT‐PCR was performed on days 14 and 21 (Figure 7a and Table S1). Genes associated with cardiac muscle development (*MYH6, MYH7, and MYL7*), cardiac muscle contraction (*TNNI3, TNNT2, and TNNI1*), ion channels (*ATP2A2, SCN5A, CACNA1C, and KCNJ2*), gap junctions (*GJA5*), cardiac transcription factors (*NKX2.5 and GATA4*), and stromal cell identity (*DDR2, ACTA2, POSTN, and VIM*) were significantly upregulated at days 14 and 21 relative to pre in situ differentiation (Figure 7a). All significant changes in gene expression and associated fold‐change values are listed in Table S1. The upregulation of cardiac transcription factors *NKX2.5* and *GATA4* confirmed early cardiac lineage commitment within the tubes, while increases in TNNI1 (troponin 1, skeletal, slow isoform), *TNNT2* (cardiac troponin T type 2), and *TNNI3* (troponin I cardiac isoform) further validated cardiomyocyte differentiation. TNNI1 was upregulated 15 110 fold at day 14 and 19 285 fold at day 21, while TNNT2 was upregulated 4 880 fold at day 14 and 7 327 fold at day 21. The lower fold‐change increase of *TNNI3* (adult cardiac isoform, 125 fold at day 14 and 75 fold at day 21) relative to *TNNI1* (foetal/slow skeletal troponin I isoform) indicates that the in situ differentiated constructs mimic an early cardiac developmental stage. The upregulation of *MYH7* (beta‐myosin heavy chain), *MYL7* (myosin light chain atrial isoform), and *MYH6* (alpha‐myosin heavy chain) at day 14 indicated that the nascent cardiac muscle was maturing, and further significant increases in *MYL2* and *MYH7* expression at day 21 suggested continued cardiac tissue maturation within the bioprinted constructs as the culture period was extended. Specifically, MYH7 increased from 400 fold at day 14 to 1 191 fold at day 21, MYL2 appeared at day 21 (285 fold), and MYL7 increased from 8 257 fold at day 14 to 14 695 fold at day 21. Genes encoding key cardiac ion channels, including *KCNJ2* (potassium ion transport), *CACNA1C* (L‐type voltage‐gated calcium), *SCN5A* (sodium influx), and *ATP2A2* (calcium regulation), increased 2‐3‐fold between days 14 and 21, indicating enhanced electrophysiological maturity with longer culture periods. Interestingly, despite the use of an adapted ventricular differentiation protocol, *GJA5* (connexin 40, atrial gap junction) expression doubled by day 21, which was consistent with positive staining for SLN. Fibroblast and stromal cell‐associated genes (*POSTN, ACTA2, DDR2, and VIM*) were also upregulated by day 14, further supporting the co‐emergence of cardiac fibroblasts alongside cardiomyocytes. By day 12, a subset of constructs exhibited spontaneous contractions (categorized as beating samples), whereas others remained non‐beating by day 21. Comparative gene expression analysis between these groups at day 21 demonstrated that the beating samples expressed higher levels of cardiac transcription factors (*NKX2.5* and *GATA4*), muscle contraction/development genes (*MYL7, MYH6, MYH7, TNNI1, TNNT2, and TNNI3*), and ion channels (*ATP2A2, SCN5A*) (Figure 7b and Table S2).

These findings collectively demonstrate that bioprinted pluripotent tissues can be guided through mesodermal and cardiac differentiation in situ via WNT pathway modulation. Unlike traditional cardiac bioprinting strategies that employ 2D pre‐differentiated iPSC‐CMs, our method induces cardiac differentiation post‐printing as the tissue undergoes shape‐morphing. By aligning differentiation and morphogenesis, this strategy more closely emulates embryonic heart development, in which cardiac progenitors and cardiomyocytes emerge within the cell‐dense, matrix‐rich, 3D microenvironment of the looping heart tube. As noted in the introduction, one prior study has reported in situ cardiac differentiation of iPSCs within bioprinted constructs [9]. The authors reported non‐uniform cardiac differentiation that was restricted to the peripheral regions of the construct, with the core areas remaining largely acellular. This spatial cellular distribution may have arisen from the use of a stiffer, covalently crosslinked collagen/GelMA‐based bioink, which could limit iPSC survival in the core regions. Although the final dimensions of our bioprinted rings were small (∼1.4 mm diameter by day 21), we observed uniform cell densities throughout the constructs, as evidenced in our cryosectioned samples (Figure 6b). The uniformity was likely facilitated by the high cell density used for bioprinting (150 million cells mL^−1^) and cell compaction resulting from the shape‐morphing process. Additionally, our embedded bioprinting approach enabled the use of a soft Matrigel bioink that supported cell proliferation and self‐organization. Cardiac progenitor specification occurred throughout the constructs, as evidenced by uniform HAND1 and IRX4 staining from the cryosections, but terminal cardiomyocyte differentiation was more restricted to peripheral regions (Figure 6b). As mentioned earlier, this suggests that the CHIR99021 and IWP‐4 small molecules could diffuse into the construct core to initiate cardiac differentiation, but that terminal cardiomyocyte differentiation was spatially restricted, likely due to limitations in oxygen and/or nutrient diffusion.

Our in situ differentiation approach resulted in the co‐emergence of cardiomyocytes and fibroblasts from a common progenitor pool within a single bioprinted construct. While co‐culture strategies have been shown to enhance iPSC‐CM differentiation [5, 40, 41], they typically involve mixing pre‐differentiated cell populations. In contrast, our in situ differentiation approach enables multiple cardiac cell types to co‐emerge within a dynamic, shape‐morphing microenvironment. The resulting cellular crosstalk during this developmental co‐emergence, which occurs when the cells are in a more developmentally plastic state, likely impacts cardiac differentiation trajectories. Positive expression for early cardiac transcription factors, such as NKX2.5 and HAND1, confirmed the progressive transition from cardiac progenitor to cardiomyocyte phenotypes within our bioprinted constructs. In traditional approaches using pre‐differentiated iPSC‐CMs, these developmental transitions occur in 2D culture conditions outside the bioprinted construct. Our in situ differentiation approach recapitulates early aspects of heart tube development, in which cardiac progenitor cells are actively differentiating into cardiomyocytes and other cardiac lineages within a 3D morphogenetic microenvironment. However, our system does not recapitulate later aspects of heart development, including heart tube looping, chamber specification, secondary heart field contributions, valve formation, and trabeculation. External mechanical stimulation could be applied to our constructs to guide later aspects of heart development, such as looping and chamber formation. For example, structural shape transformations produce mechanical stresses in the developing heart tube that coincide with cardiac progenitor‐to‐cardiomyocyte transitions [16, 17]. Interestingly, it has been demonstrated that early‐stage iPSC‐derived cardiac tissue is more responsive to electromechanical conditioning than late‐stage tissue [42]. This suggests that the immature phenotype of our in situ differentiated bioprinted constructs may be advantageous for enhancing maturation via externally applied mechanical stimulation, and future work will explore this.

A limitation of our approach is the spatial restriction of terminal cardiomyocyte differentiation to peripheral regions of the constructs, with cells in core regions appearing to persist in a cardiac progenitor phenotype, likely due to limitations in oxygen and nutrient diffusion. Integrating perfusable vascular channels could enhance oxygen and nutrient delivery to core regions, potentially enabling more spatially uniform terminal cardiomyocyte differentiation throughout the constructs. Differentiation efficiency could potentially be improved by optimizing the cardiac induction protocol. For example, the exogenous addition of Activin‐A and BMP‐4 alongside CHIR99021 during mesoderm induction has been shown to improve the efficiency of cardiac‐specific mesoderm specification [43, 44]. CHIR99021‐mediated Wnt activation induces mesodermal specification through downstream Activin‐A and BMP signalling, and temporal supplementation of these factors exogenously could result in more efficient and spatially uniform cardiomyocyte differentiation within our 3D constructs. The optimal CHIR99021 concentration is often narrow and requires optimization for each iPSC line. We observed that 6 and 7 µM CHIR99021 supported successful in situ cardiomyocyte differentiation in our bioprinted constructs, but higher concentrations that support optimal 2D differentiation of the same iPSC line [5] were ineffective in the 3D setting. More systematic optimization of CHIR99021 concentration, exposure duration, and timing could be explored to enhance the efficiency of our in situ differentiation approach. Bioink composition may also influence differentiation outcomes. We employed Matrigel for the cardiac differentiation experiments as it supported high iPSC viability and post‐printing shape‐morphing behavior (Figure 2a, b). Collagen type 1 bioinks, which are more commonly used for iPSC‐cardiomyocyte bioprinting, supported high iPSC viability, but the bioprinted constructs did not undergo shape‐morphing (Figure 2a, b). Matrigel has limitations for translational applications due to its batch‐to‐batch variability and tumor‐derived origin, and future work will therefore explore bioinks formulated with defined matrices, such as laminin or vitronectin, which support iPSC culture in 2D. Fibronectin could also be explored, as it has been shown to enhance cardiomyocyte differentiation [45].

Scaling to clinically relevant tissue dimensions remains an important challenge that we have not yet addressed with our in situ differentiation approach and will require integrating perfusable vascular networks to enhance nutrient and oxygen delivery to core regions. Interestingly, our cryosection data highlighted a potential advantage of our in situ approach in terms of scalability. As noted above, robust staining for cardiac progenitor markers was observed throughout the construct thickness, whereas cTnT staining was more restricted to peripheral regions (Figure 6b). The limited progenitor‐to‐cardiomyocyte maturation in deeper tissue regions may be due to oxygen diffusion limitations that restrict this transition. This suggests that bioprinting iPSCs or cardiac progenitors may offer an inherent advantage over bioprinting more mature differentiated cardiomyocytes for scaling to larger tissue dimensions. Progenitor cells primarily rely on glycolytic metabolism and may be better adapted to survive in hypoxic regions of larger constructs before functional perfusion is established [46, 47, 48]. In contrast, mature cardiomyocytes depend on oxidative phosphorylation and are therefore more susceptible to cell death in poorly perfused core regions. Future work will explore whether combining bioprinted cardiac progenitors with perfusable vascular networks can enhance overall cell viability and the uniformity of cardiac progenitor‐to‐cardiomyocyte differentiation throughout the tissue volume.

## 3 Conclusion

This study presents a novel developmentally inspired strategy for bioprinting human heart tissue by directing the in situ cardiac differentiation of iPSCs within shape‐morphing constructs. The embedded bioprinting of Matrigel bioinks containing high‐density suspensions of iPSCs into granular support baths, followed by post‐printing cellular condensation, generated pluripotent, cell‐dense tissues capable of undergoing shape‐morphing. Support bath mechanics were optimized to maintain pluripotency, with softer formulations minimizing spontaneous differentiation while allowing shape‐morphing. Temporal modulation of WNT signalling guided mesodermal and cardiac differentiation within the shape‐morphing constructs, generating nascent human heart tissues in which cardiomyocytes and fibroblasts co‐emerged from a common progenitor pool. By aligning shape‐morphing with in situ mesoderm and cardiac lineage specification, this approach recapitulates early aspects of cardiac development. Overall, this developmentally inspired approach represents an alternative to conventional 2D pre‐differentiation paradigms and offers a promising pathway for bioprinting nascent, multicellular heart tissues with an endogenous capacity for structural maturation.

## 4 Experimental Section

### 4.1 Human Induced Pluripotent Stem Cell (iPSC) Culture, Maintenance, and Expansion

Human iPSCs (A18945, Fisher Scientific) were seeded on Corning Matrigel (354277, Fisher Scientific) coated 6‐well plates. The Matrigel coating solution was prepared by diluting Matrigel (2%) in Gibco DMEM F‐12 GlutaMAX supplement medium (10565018, Fisher Scientific), and the plates were coated for 1 h at room temperature, followed by incubation at 37°C for 20 min. 5 µm ROCK Inhibitor Y‐27632 (72304, STEMCELL Technologies) was added to the culture medium during thawing to enhance cell survival. iPSCs were detached using Accutase (07920 or 07922, STEMCELL Technologies) for 5–7 min in incubation at 37°C. Detached cells were centrifuged at 300G for 5 min, and the cell pellet was gently dissociated to seed them either on coated well plates or embedded in ink. iPSCs were maintained in a 1:1 ratio of mTeSR Plus (100–1130, STEMCELL Technologies) and Gibco Essential 8 medium (A2858501, Fisher Scientific) for the first day after thawing and passaging, followed by daily changes of Gibco Essential 8 medium until the cells reached 80%–90% confluency. For iPSC bioprinting, cells were used between passages 9–12. ROCK inhibitor (10 µm concentration) was added to the Gibco Essential 8 medium for 24 h post‐bioprinting to enhance cell survival. For the first three days, the media was changed daily; after three days, it was changed on alternate days.

### 4.2 Pre‐Formed Embryoid Bodies and Bioprinting

iPSCs were passaged once the cells reached 80%–90% confluency. The cell suspension (2.5 million cells per well) was reseeded in an AggreWell 400 6‐well plate (34425, STEMCELL Technologies) after passaging. Before seeding, the plate was coated with an anti‐adherence solution (07010, STEMCELL Technologies) for 5 min and centrifuged at 1300G for 5 min. This was followed by rinsing with warm culture medium once and then storing in an incubator with culture medium unless the cells were passaged. After seeding the cells, the plate was centrifuged at 100G for 3 min. For the first day, a 1:1 ratio of mTeSR plus medium and Gibco Essential 8 medium was added (total 4 mL each well), followed by Essential 8 medium the next day. Pre‐formed EB were aspirated using a cut pipette after two days and encapsulated in a Matrigel‐based bioink for bioprinting. ROCK inhibitor (10 µm) was added for the first 24 h, followed by Gibco Essential 8 medium. For the first three days, the media was changed daily; after three days, it was changed on alternate days.

### 4.3 In Situ Differentiation to Cardiac Tissue

Bioprinted iPSC tissues were maintained in Gibco Essential 8 medium with daily media changes and gradual agarose removal via media exchange over three days. The agarose was diluted through successive additions of media, and the diluted agarose was gradually removed until it was completely replaced by the media. As mentioned above, ROCK inhibitor (10 µm) was added to the Gibco Essential 8 medium for 24 h post‐bioprinting to enhance cell survival. On day 3, iPSC‐laden tissue (from passages 9 and 11) was directed toward the mesoderm lineage by adding 6–7 µm CHIR99021 (72054, STEMCELL Technologies) and removing the agarose support bath, which was considered day 0 of differentiation. This was followed by 1 µm CHIR99021 on day 1, 5 µm IWP‐4 (72554, STEMCELL Technologies) on day 3. These small molecules were added to Gibco RPMI 1640 medium (11875085, Fisher Scientific) supplemented with Gibco B27 minus insulin (2%) (A1895601, Fisher Scientific). This was followed by medium change without any small molecules on day 5 of differentiation. Next, the culture medium was changed to RPMI 1640 supplemented with Gibco B27 supplement (2%) (17504044, Fisher Scientific) and 1% penicillin‐streptomycin on day 8 and changed every 2 days until day 14.

### 4.4 Bioink Preparation

Type I bovine atelocollagen (PureCol 3 mg mL^−1^ from Advanced Biomatrix 5005) was neutralized according to the manufacturer's instructions to adjust the pH to ≈7.2. The final concentration after neutralization was 2.4 mg mL^−1^, and the collagen was then diluted to a final concentration of 0.96 mg mL^−1^ for cell encapsulation and printing (by volume; 40% collagen, 60% media and cells). The bioink was stored in an icebox to prevent gelation before cell encapsulation and bioprinting. Corning Matrigel (354277, Fisher Scientific) of dilution factor 260µl (Lot no. 12224005) was used for all experiments. According to the manufacturer's details, the typical protein concentration of the Corning Matrigel matrix is 8–11 mg/mL ^−1^. For the preparation of our Matrigel bioink, the final Matrigel protein concentration was diluted to approximately 3.2–4.4 mg mL^−1^ (by volume; 40% Matrigel, 60% media and cells). To prepare the composite collagen and Matrigel bioinks, the final concentrations of collagen and Matrigel were 0.6 mg mL^−1^ and 2–2.75 mg mL^−1^, respectively (by volume; 25% collagen, 25% Matrigel, 50% media and cells).

### 4.5 Agarose Support Bath Fabrication

Agarose type I, low EEO was purchased from Sigma–Aldrich (A6013). Agarose microparticles were prepared using a shearing technique to form a viscoelastic suspension bath with varied stiffness [49, 50]. Briefly, a 0.5 wt.% agarose solution (1X PBS) was autoclaved at 121°C, and then the molten solution was allowed to cool at room temperature for 3 h under stirring at 700 rpm. This protocol results in the formation of microparticles via shearing as the agarose solidifies. The agarose microparticle solution was maintained under sterile conditions and stored for up to 3 months at 4–7°C. Prior to iPSC and iPSC‐CM bioprinting, agarose particles were centrifuged at 900G for 10 min and resuspended in the same volume of E8 medium and RPMI 1640 supplemented with Gibco B27 supplement (2%) and 1% penicillin‐streptomycin.

### 4.6 3D Bioprinting Setup and Embedded Bioprinting

Bioprinting experiments were performed using an in‐house designed bioprinter, Bioframe (Figure S1b). Bioframe is a desktop core XY 3D printer that supports a high degree of modifications for specific tasks. It features swappable tool heads and print beds (Figure S1d), a large vertical printing range, and runs on the open‐source 3D printer firmware, Klipper, allowing for the integration of additional electronic components, such as cameras and light sources. For these experiments, the Bioframe was configured with a deep vessel for printing tall structures and a Puredyne progressive cavity pump extruder (Figure S1c). The Bioframe was placed inside a biosafety hood to maintain sterile conditions (Figure S1e). Models for printing were prepared using computer‐aided design (CAD) software, Autodesk Inventor, and G‐code for tool path planning of the prints was generated using Prusa Slicer on a custom profile for the Bioframe (Figure S1f). This profile was tuned for use with 27G needles, where the desired extrusion should be of a similar width to the needle, and the printer should operate at a speed of 1 mm/s. Live adaptation was performed on the prints via the Klipper user interface installed on the Bioframe, by adjusting the extrusion factor to 120%–150% of the extrusion values prescribed by the slicer in order to achieve optimal print quality. During bioprinting, the material temperature in the extruder was maintained at 6–7°C using a Puredyne temperature control unit, and post‐bioprinting, the constructs were incubated immediately at 37°C. High glass bottom µ‐Dishes (35 mm, 81158, ibidi) were used to culture the bioprinted constructs. These dishes enabled imaging of the tissue within the support bath during the culture period using an inverted microscope. The culture dishes were adapted to contain two compartments separated by a solid agarose divider (2 wt.%). Culture medium was added to one compartment, and an agarose suspension bath was added to the second compartment [49]. The agarose divider allowed the diffusion of the culture medium into the bioprinted constructs suspended in the support bath. The agarose suspension bath supported the bioprinted tissue for the first three days of culture and was then removed for longer culture as well as for in situ differentiation (except for the bath stiffness study, where the bath was not removed until day seven). The media was changed daily for the first three days and then on alternate days.

### 4.7 Rheological Characterization

The rheological properties of the varying bath stiffness were characterized using rotational shear rheometry (25 mm diameter, gap 1 mm; Anton Paar MCR 302) at room temperature. Storage and loss modulus were measured separately as a function of shear strain (0.0001^−1^ ‐1^−1^).

### 4.8 Microscopy Analysis of Bioprinted Constructs

A brightfield microscope (Dinocapture 2.0) was used to visualize live samples during the culture period. Images of the bioprinted constructs were captured at different time points, and FIJI software was used to measure the shape change relative to day 0. For ring geometries, shape‐morphing was quantified as the change in ring outer diameter (∅) over time using the following formula: shape‐morphing (%) = (∅_*Day* 0_  − ∅_*Day* 7_)/(∅_*Day* 0_). For beam geometries, shape‐morphing was quantified as the change in beam length (*L*) over time using the following formula: shape‐morphing (%) = (*L*_*Day* 0_  − *L*_*Day* 7_)/(*L*_*Day* 0_). Inverted confocal microscopy was used to image bioprinted samples containing fluorescein isothiocyanate‐conjugated collagen and cell dye. Invitrogen CellTracker dyes (C34552:15 µM, Fisher Scientific) were used to track shape changes. Bioprinted tissues were visualized live on the day of bioprinting (day 0), and fluorescence images were obtained using multi‐channel confocal microscopy (Andor benchtop BC43).

### 4.9 Live/Dead Staining

Live/dead staining was performed by treating samples with 2 µm Calcein AM (11564257, Fisher Scientific) and 2 µm ethidium homodimer (10184382, Fisher Scientific) in 1X PBS, followed by 30 min incubation at 37°C. The samples were imaged using an Andor benchtop BC43 confocal microscope. This study quantified the percentage of live cell area (Viability = Live cell area/ (live + dead cell area) as it was difficult to count individual live cells from these images due to the high levels of cell‐cell contact.

### 4.10 Phalloidin TRITC Staining

To visualize the actin cytoskeleton, fixed bioprinted constructs were stained with 0.05 mg mL^−1^ phalloidin tetramethylrhodamine B isothiocyanate (P1951, Merck) for 2 h. The constructs were then washed twice with 1X PBS, followed by staining with Fluoroshield DAPI for 20 min at room temperature, and the samples were visualized using confocal microscopy.

### 4.11 Immunofluorescence Staining of the Constructs and Cryosections

Samples for immunofluorescence staining were fixed with 10% neutral buffered formalin solution overnight at 4°C and stored in DPBS the following day until staining. A subset of the samples was embedded in OCT (optimal cutting temperature) compound and stored in −80°C prior to cryosectioning. The frozen samples were sectioned into 10 µm slices by a cryostat and collected onto Superfrost Plus glass slides. Prior to staining, the sections were air‐dried at room temperature for 30–60 min and stored at −20°C. Both the construct and the cryosections were then permeabilized with Triton‐X 100 (0.2%) in PBS and 2% BSA for 45 min and then blocked using 2% BSA in PBS for 1 h. Primary antibody (1:200 dilution in blocking buffer) staining was then carried out overnight at 4°C, followed by three washes with PBS and then adding the secondary antibody for 2 h (1:200 dilution in blocking buffer). Samples were washed thrice before counterstaining with Fluoroshield DAPI (F6057, Merck) for 20 min. The cryosections were then enveloped with coverslips, and the non‐sectioned constructs were kept in PBS and imaged directly. Imaging was performed using an Andor benchtop BC43 confocal microscope, and the images were processed using ImageJ software. All primary and secondary antibodies are listed (Section S3). Color‐based quantification of the immunofluorescence images was performed by converting images from the RGB to the HSV color space, followed by threshold‐based segmentation using predefined ranges for each target color. Specifically, green was defined as H = 35–85, S = 50–255, V = 50–255; blue as H = 90–130, S = 50–255, V = 50–255; and red using two hue intervals to account for circularity of the HSV space (H = 0–10 and 170–179, with S = 50–255 and V = 50–255). Binary masks were generated for each color and denoized using median filtering. Quantitative metrics were then derived by summing the pixel counts per color class and expressing them as relative proportions and inter‐color ratios with respect to the total detected area. All analyzes were implemented in Python (version 3.12.10), using libraries including NumPy, OpenCV‐python, and Scikit‐image.

### 4.12 RNA Isolation and qPCR

The PureLink RNA Mini Kit (10307963, Invitrogen) was used to isolate RNA from 2D iPSC, bioprinted iPSC, and iPSC‐CM samples. cDNA preparation was carried out using the Invitrogen SuperScript IV VILO Master Mix (15523145) or RT2 First Strand Kit (330404, Qiagen) and processed in a Veriti Thermal Cycler. RT‐PCR was performed using the Applied Biosystems TaqMan Fast Advanced Master Mix for qPCR (11380912) or RT2 SYBR Green ROX qPCR Mastermix (330522, Qiagen) for a custom‐designed RT2 PCR Array 96‐well plate (330171, Qiagen), maintaining a 10% excess amount for the qPCR run. QuantStudio 5 was used to run the qPCR, and data analysis was performed in Excel using the CT value. Heatmaps were plotted in OriginPro 2023 software based on the fold‐change value calculated using the CT value, considering GAPDH as the housekeeping gene for all studies. Further details on the genes can be found in Section S3.

### 4.13 Calcium Imaging

Calcium flux dynamics were assessed by treating the bioprinted constructs with 4 µm Cal 520 for 90 min at 37°C accompanied by 0.04% pluronic to amplify cellular uptake. The dye was prepared in cardiomyocyte maintenance media. Following incubation, the construct was washed three times with the cardiomyocyte maintenance media, and the fluorescence videos were recorded at 10 fps using the Andor benchtop BC43 confocal microscope at 10X magnification at 37°C. The fluorescence intensity traces from the videos were quantified in ImageJ using three ROIs, and the graphs were plotted in GraphPad Prism, normalizing the signal to background fluorescence (F/F_0_). The calcium transient duration was measured as the time from peak depolarization (max fluorescence signal) to 90% repolarization (90% reduction in fluorescence signal from peak).

### 4.14 Contraction Imaging

Videos of the bioprinted constructs were captured under brightfield illumination using a Leica DMi inverted microscope at 10x magnification for 30 s on day 14 at room temperature. For drug response analysis, following baseline recording, 1 µm isoproterenol was added to the culture media, and videos were captured after a 30 min incubation at 37°C (Movie S2). The Musclemotion macro in ImageJ was used to determine the contraction amplitude and peak‐to‐peak time [51]. GraphPad Prism software was used for plotting the contraction graphs.

### 4.15 Statistical Analysis

All experimental data were compiled and analyzed using Microsoft Excel. All graphs are presented as means with standard deviation, along with sample numbers denoted in each graph and figure legends. The reported sample sizes (n‐numbers) in the figure legends denote biologically independent samples in which undifferentiated iPSCs were bioprinted into separate constructs, and the in situ differentiation process occurred independently within each construct. Statistical analyzes were performed using GraphPad Prism 10 software. Unpaired t‐test, two‐way or one‐way ANOVA tests were used depending on the number of independent variables within the experiment, and Tukey's multiple comparison test was used to compare differences between means. P values are described as follows: ns denotes not significant, ^*^ *p* <0.05, ^**^ *p* < 0.01, ^***^ *p* < 0.001, ^****^ *p* < 0.0001.

## Conflicts of Interest

The authors declare no conflict of interest.

## Supporting information




**Supporting File 1**: advs75760‐sup‐0001‐SuppMat.docx.

**Supporting File 2**: advs75760‐sup‐0002‐MovieS1.avi.

**Supporting File 3**: advs75760‐sup‐0003‐MovieS2.avi.

## Data Availability

The data that support the findings of this study are openly available in Zenodo at https://doi.org/10.5281/zenodo.20121878, reference number 20121878.
