# Supplementary material for: Developmentally Inspired Bioprinting of Nascent Multicellular Human Heart Tissue Through in Situ Differentiation and Morphogenesis of iPSCs
Source: Adv Sci (Weinh). 2026 May 20:e22241. Online ahead of print. doi: 10.1002/advs.202522241 (PMC13336075; doi:10.1002/advs.202522241)
Supplement: Supplementary file 1 — Supporting File 1: advs75760‐sup‐0001‐SuppMat.docx. [file ADVS-9999-e22241-s003.docx]

Supporting Information S1 – Additional Table

**Table S1.1:** Overview of genes that were significantly upregulated at Days 14 and 21 within *in situ* differentiated constructs relative to before *in situ* differentiation. Fold change values represent gene expression values at Day 14/21 relative to before *in situ* differentiation, with GAPDH as the housekeeping gene. At Day 21, the constructs were separated into beating and non-beating groups. Statistical analysis was based on an unpaired t-test, and significant genes were selected based on p< 0.05. All gene expression fold-change values are presented as heatmaps in Figure 7. A statistical comparison between the beating and non-beating samples is presented in Table S1.2.

| **D14 Beating** | **Fold change** | **D21 Beating** | **Fold change** | **D21**  **Non-Beating** | **Fold change** |
| --- | --- | --- | --- | --- | --- |
| POSTN | 422.85 | VIM | 44.63 | VIM | 50.00 |
| ACTA2 | 3108.47 | POSTN | 2846.21 | POSTN | 61.52 |
| DDR2 | 27.35 | ACTA2 | 5844.57 | ACTA2 | 198.91 |
| NKX2-5 | 4420.90 | DDR2 | 42.99 | DDR2 | 6.32 |
| GATA4 | 775.21 | NKX2-5 | 4314.28 | TNNI1 | 34.64 |
| TNNI1 | 15109.84 | GATA4 | 997.77 | TNNI3 | 4.59 |
| TNNT2 | 4880.00 | TNNI1 | 19284.87 | GJA1 | 3.87 |
| TNNI3 | 125.93 | TNNT2 | 7327.01 | GJA5 | 3.90 |
| GJA5 | 77.89 | TNNI3 | 75.42 | CACNA1C | 5.35 |
| KCNJ2 | 7.87 | GJA5 | 142.92 | ATP2A2 | 2.84 |
| CACNA1C | 35.21 | KCNJ2 | 16.72 | MYH7 | 16.20 |
| SCN5A | 5.49 | CACNA1C | 121.46 |  |  |
| ATP2A2 | 5.94 | SCN5A | 11.37 |  |  |
| MYL7 | 8257.74 | ATP2A2 | 8.91 |  |  |
| MYH7 | 400.26 | MYL7 | 14695.61 |  |  |
| MYH6 | 16185.97 | MYL2 | 285.22 |  |  |
|  |  | MYH7 | 1191.22 |  |  |
|  |  | MYH6 | 20566.46 |  |  |

**Table S1.2:** Overview of genes that were significantly upregulated in ‘beating’ relative to ‘non-beating’ constructs at day 21. As per Table S1.1, fold change values represent gene expression values at Day 21 relative to before *in situ* differentiation, with GAPDH as the housekeeping gene. Statistical analysis was based on an unpaired t-test, and significant genes were selected based on p< 0.05. All gene expression fold-change values are presented as heatmaps in Figure 7.

| **Gene** | **D21 Non-beating (fold change)** | **D21 Beating (fold change)** |
| --- | --- | --- |
| ACTA2 | 198.910 | 5844.570 |
| DDR2 | 6.321 | 42.987 |
| NKX2-5 | 22.511 | 4314.276 |
| GATA4 | 14.568 | 997.767 |
| TNNI1 | 34.637 | 19284.866 |
| TNNT2 | 11.694 | 7327.005 |
| TNNI3 | 4.590 | 75.416 |
| GJA5 | 3.900 | 142.917 |
| SCN5A | 2.942 | 11.372 |
| ATP2A2 | 2.843 | 8.908 |
| MYL7 | 5.987 | 14695.608 |
| MYH7 | 16.198 | 1191.218 |
| MYH6 | 11.731 | 20566.457 |

**Supporting Information S2 – Additional Figures**

**
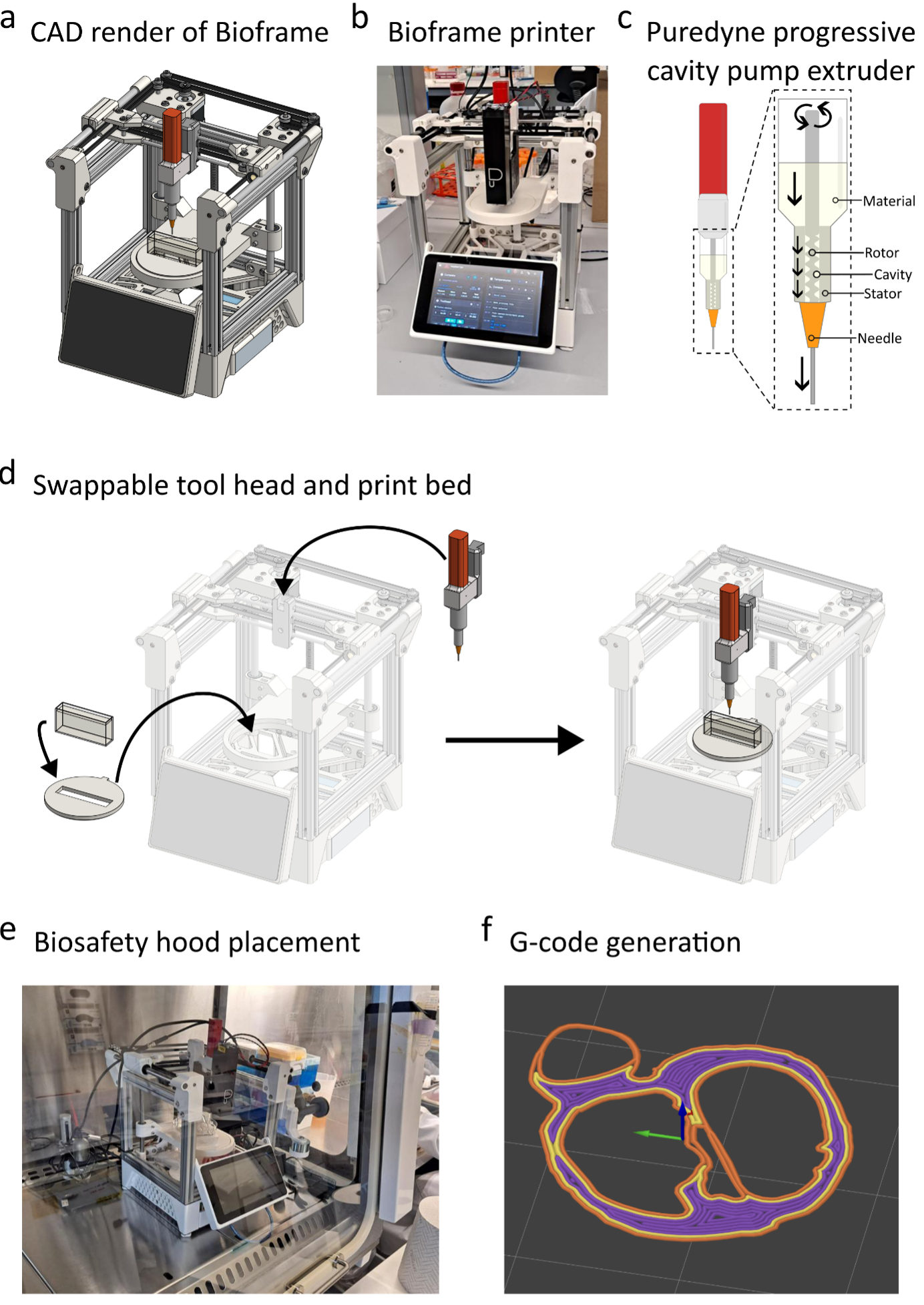
**

**Supplementary figure 1 Custom-built bioprinting hardware specifications and operation: a)** ‎CAD render of Bioframe bioprinter. **b)** Final assembled Bioframe featuring core XY kinematics, temperature-controlled extruder, and Klipper firmware with Mainsail user interface. **c)** Puredyne progressive cavity pump extruder used for bioprinting. **d)** Mechanism for ease of changing tool heads and printbeds on the Bioframe. **e)** Installation of the Bioframe unit in the biosafety hood for sterile printing conditions. **f)** Toolpath rendering from the generated G-code using PrusaSlicer.


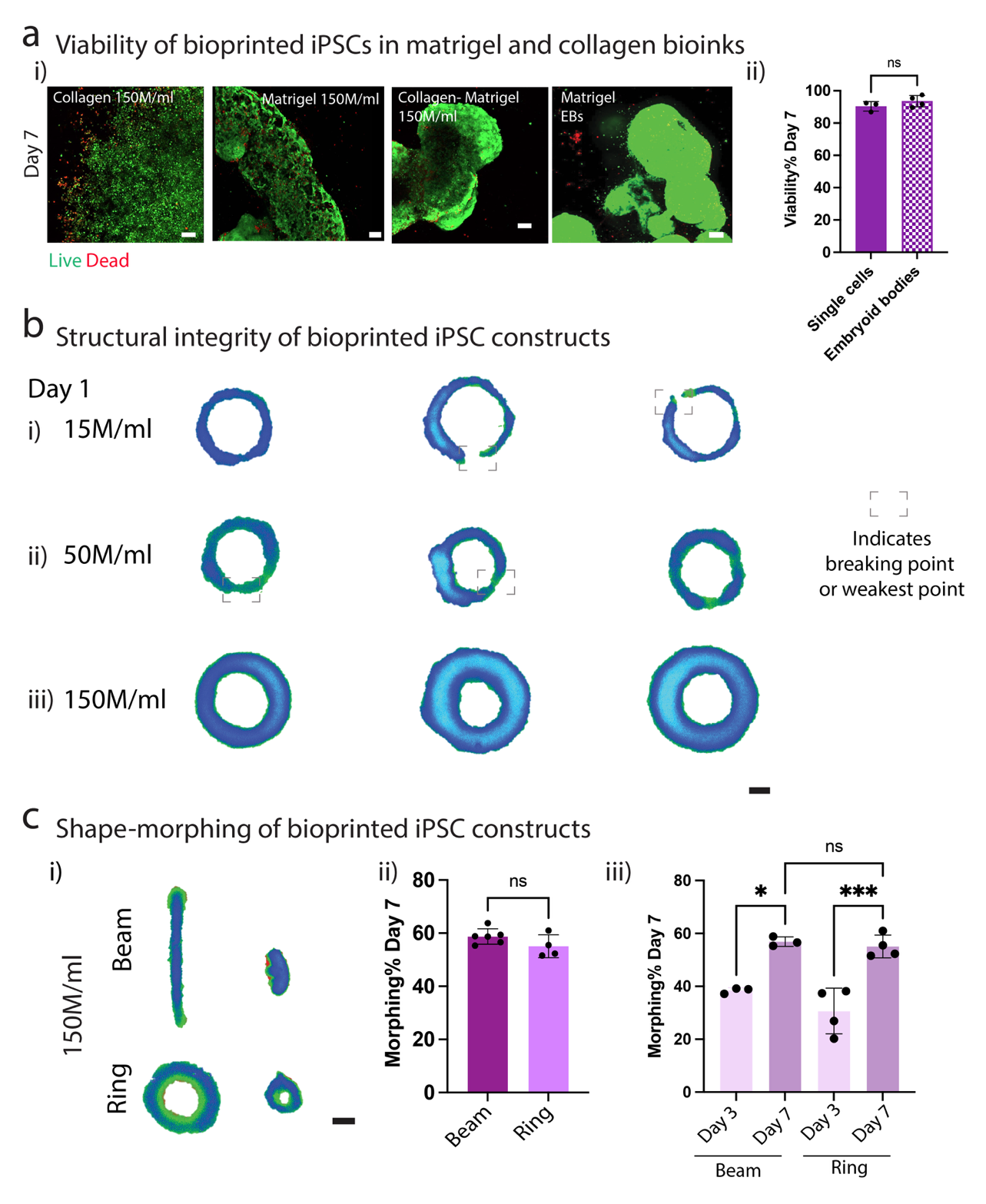


**Supplementary figure 2 Optimising viability and printability of bioprinted iPSCs: a)** i) Confocal images of live-dead viability staining for different bioink compositions and cell entities (scale bar 100 µm), ii) Quantitative analysis demonstrating the viability of bioprinted tissue on day 7, varying the cell entity within bioink (single cells vs. embryoid bodies). All biological replicates (n=3/4) and an unpaired t-test was performed, where ns denotes no significance. **b)** Printability of bioprinted constructs was evaluated through brightfield images post 24h bioprinting to identify the weak points or breakage along the fabricated ring for i) 15, ii) 50, and iii) 150 million cells ml^-1^ (scale bar 1 mm). **c)** i) Brightfield images highlighted the tissue shape-morphing in culture for beam and ring constructs with 150 million cells mL^-1^ (scale bar 1 mm). ii) Quantitative analysis demonstrated the extent of shape-morphing in beam and ring constructs over seven days of culture. iii) The Beam and Ring constructs demonstrated a significant extent of shape-morphing from day 3 to day 7, which was analysed quantitatively from brightfield images using Fiji software. All biological replicates (n=4-6), one-way ANOVA was performed where ns denotes not significant, * denotes p <0.05, and *** denotes p< 0.001.


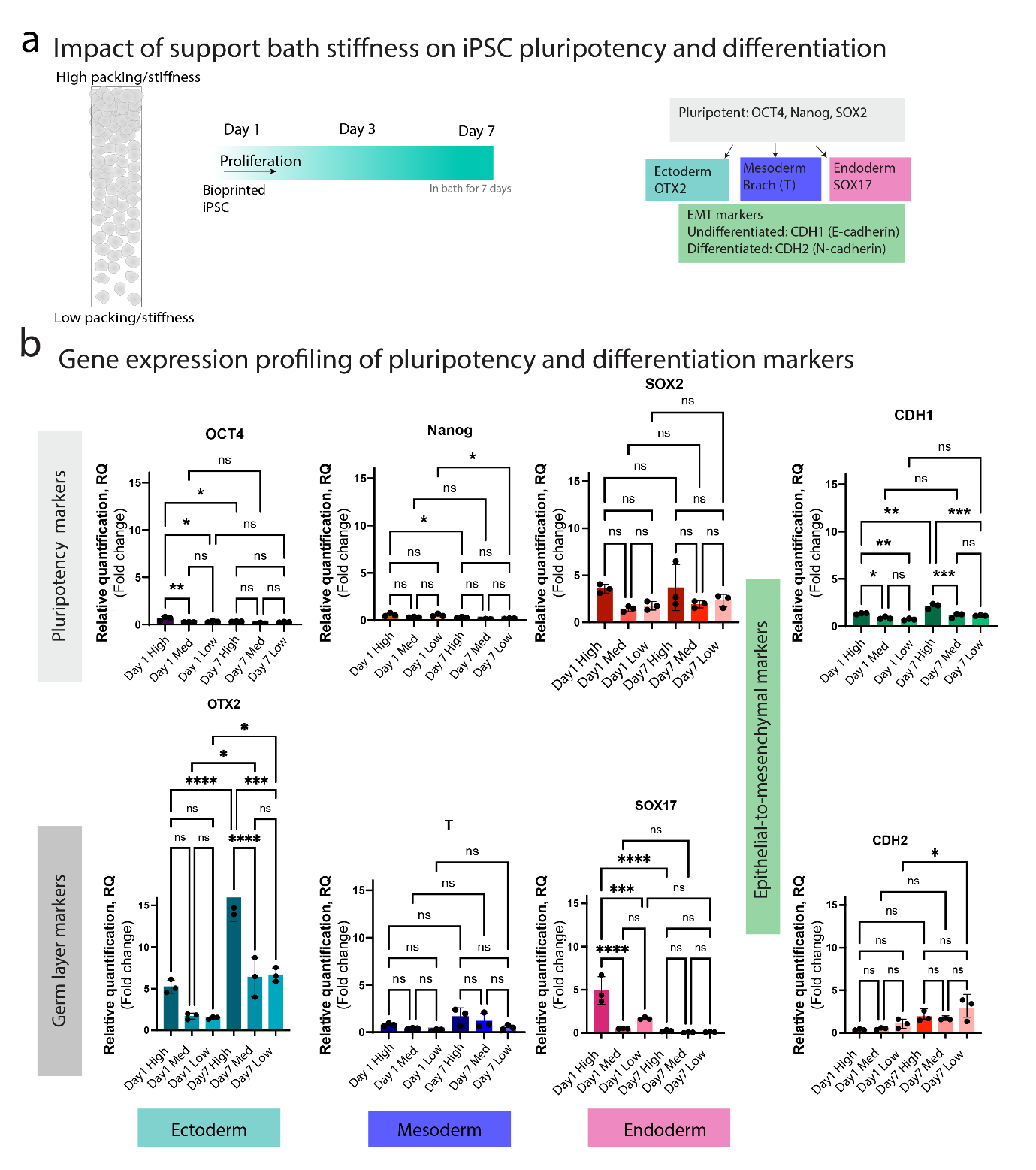


**Supplementary figure 3** **Pluripotency, germ layer, and EMT expression within bioprinted morphing iPSCs tissue constructs in a granular support bath: a)** Schematic diagram representing the variation in packing density of the support bath (high, medium, and low) at the indicated time points selected for the evaluation of pluripotency, germ layer, and EMT markers using qRT-PCR. **b)** Plot of individual genes representing pluripotency (OCT4, Nanog, SOX2), germ layer (Ectoderm OTX2, Mesoderm T, and endoderm SOX17), and epithelial-mesenchymal transition (EMT) (E-cadherin CDH1 and N-cadherin CDH2) markers while the bioprinted tissue undergoes shape-morphing. Biological replicates n=3, one-way ANOVA was performed with Tukey’s multiple comparison test, where ns denotes not significant, * denotes p< 0.05, ** denotes p < 0.01, *** denotes p < 0.001, and **** denotes p < 0.0001.


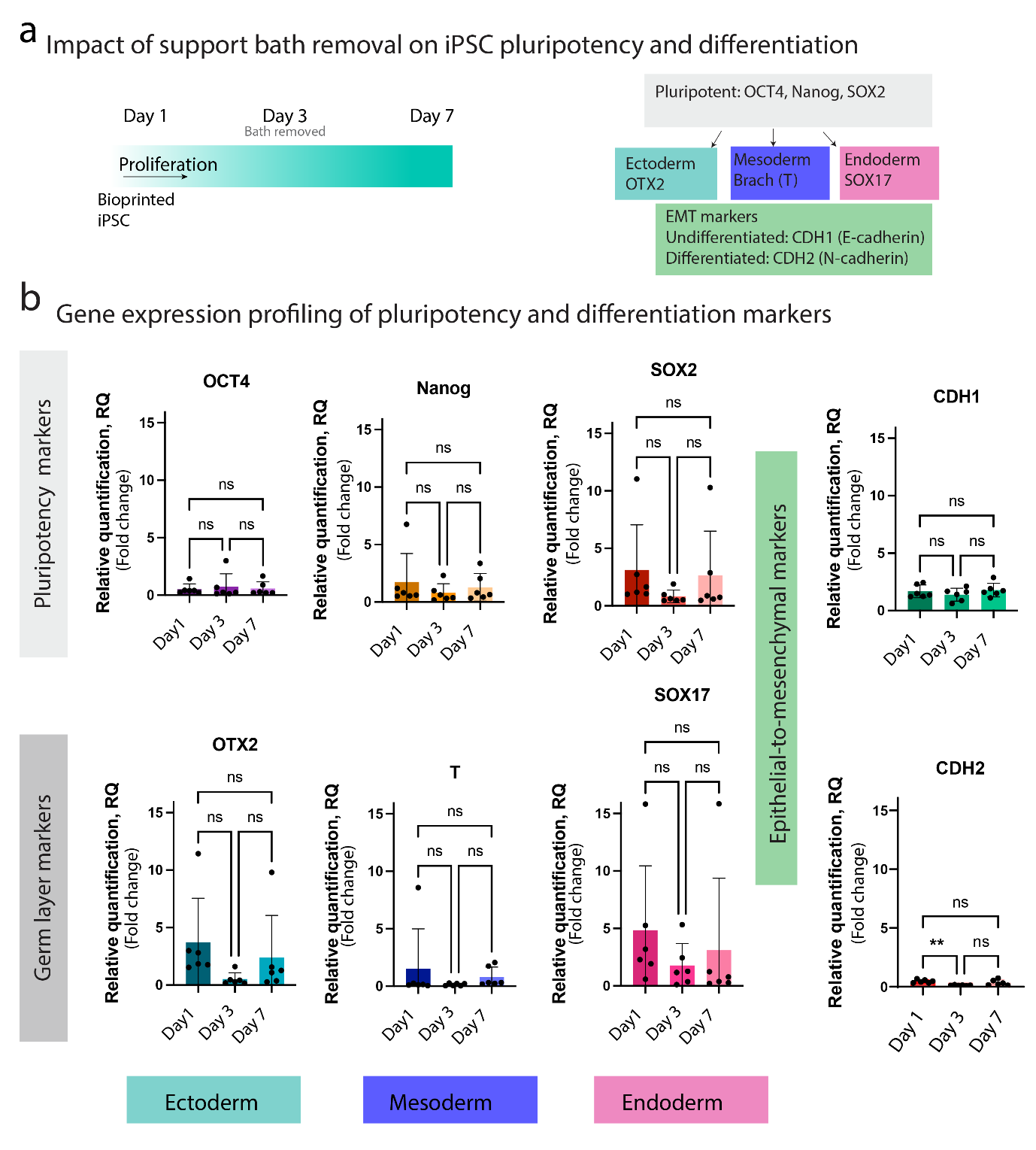


**Supplementary figure 4 Expression of pluripotency, germ layer, and EMT within bioprinted morphing iPSCs tissue constructs: a)** Schematic diagram indicating the time points selected for investigating the fold change of pluripotency, germ layer, and EMT expression (right). The panel of genes employed for qRT-PCR (left). **b)** Plot of individual gene representing pluripotency (OCT4, Nanog, SOX2), germ layer (Ectoderm (OTX2), Mesoderm (T) and endoderm (SOX17)) and EMT (E-cadherin CDH1 and N-cadherin CDH2) markers while the bioprinted tissue undergoes shape-morphing. Biological replicates n=6, one-way ANOVA performed with Tukey’s multiple comparison test, where ns denotes not significant, and ** denotes p < 0.01.


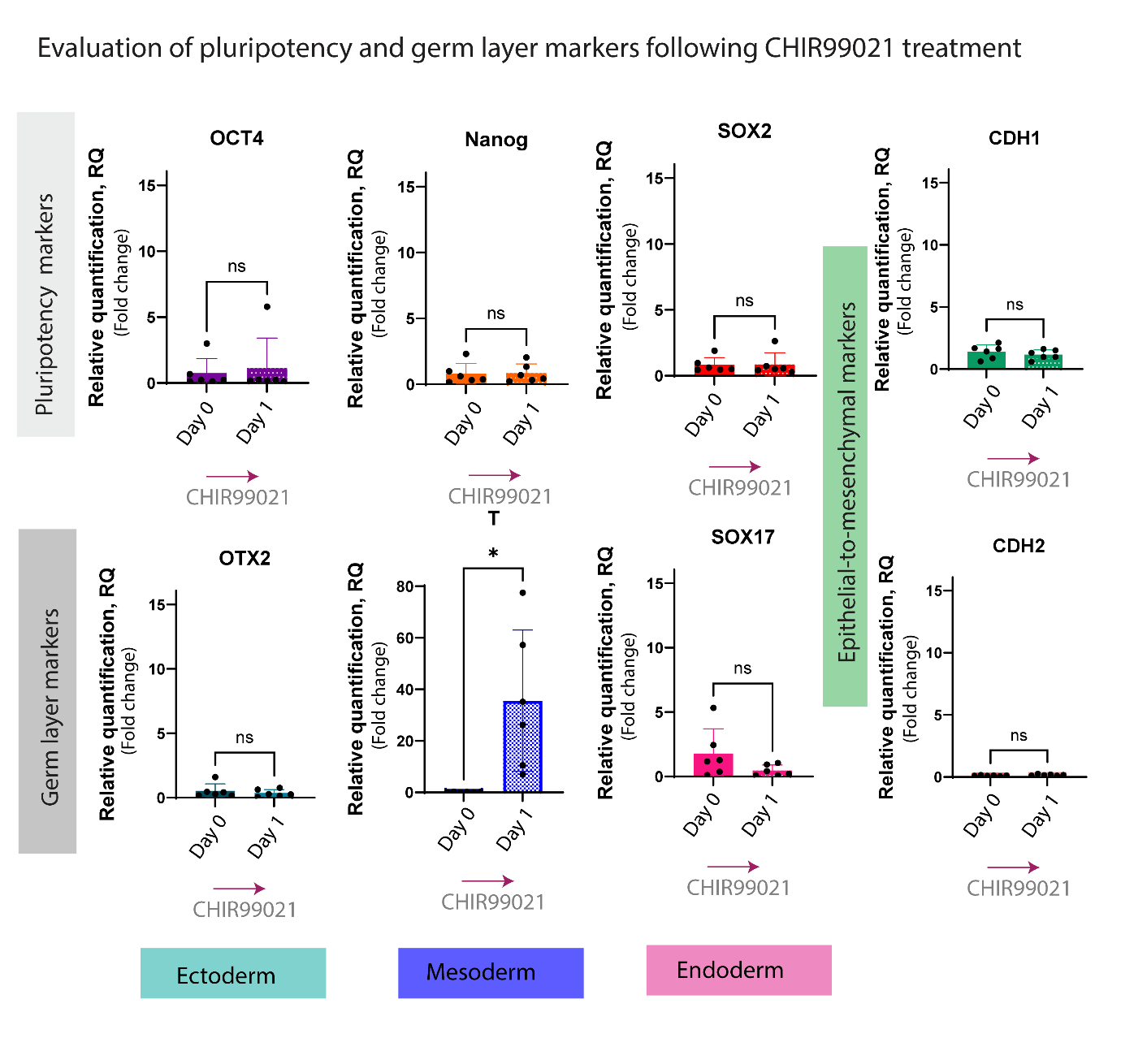


**Supplementary figure 5 Evaluation of pluripotency, germ layer, and EMT expression within bioprinted constructs upon mesoderm induction by WNT activation:** Plot of individual genes associated with pluripotency (OCT4, Nanog, SOX2), germ layer (Ectoderm OTX2, Mesoderm T, and endoderm SOX17), and EMT (E-cadherin CDH1 and N-cadherin CDH2) expression on mesoderm induction by activating the WNT pathway using CHIR99021, a small molecule that inhibits the GSK3 pathway and initiates mesoderm specification. Brachyury (T), which is related to mesoderm induction, showed significant upregulation after 24h post-CHIR99021 addition. All biological replicates (n = 6) and one-way ANOVA were performed with Tukey’s multiple comparison test, where ns denotes not significant, * denotes p < 0.05.


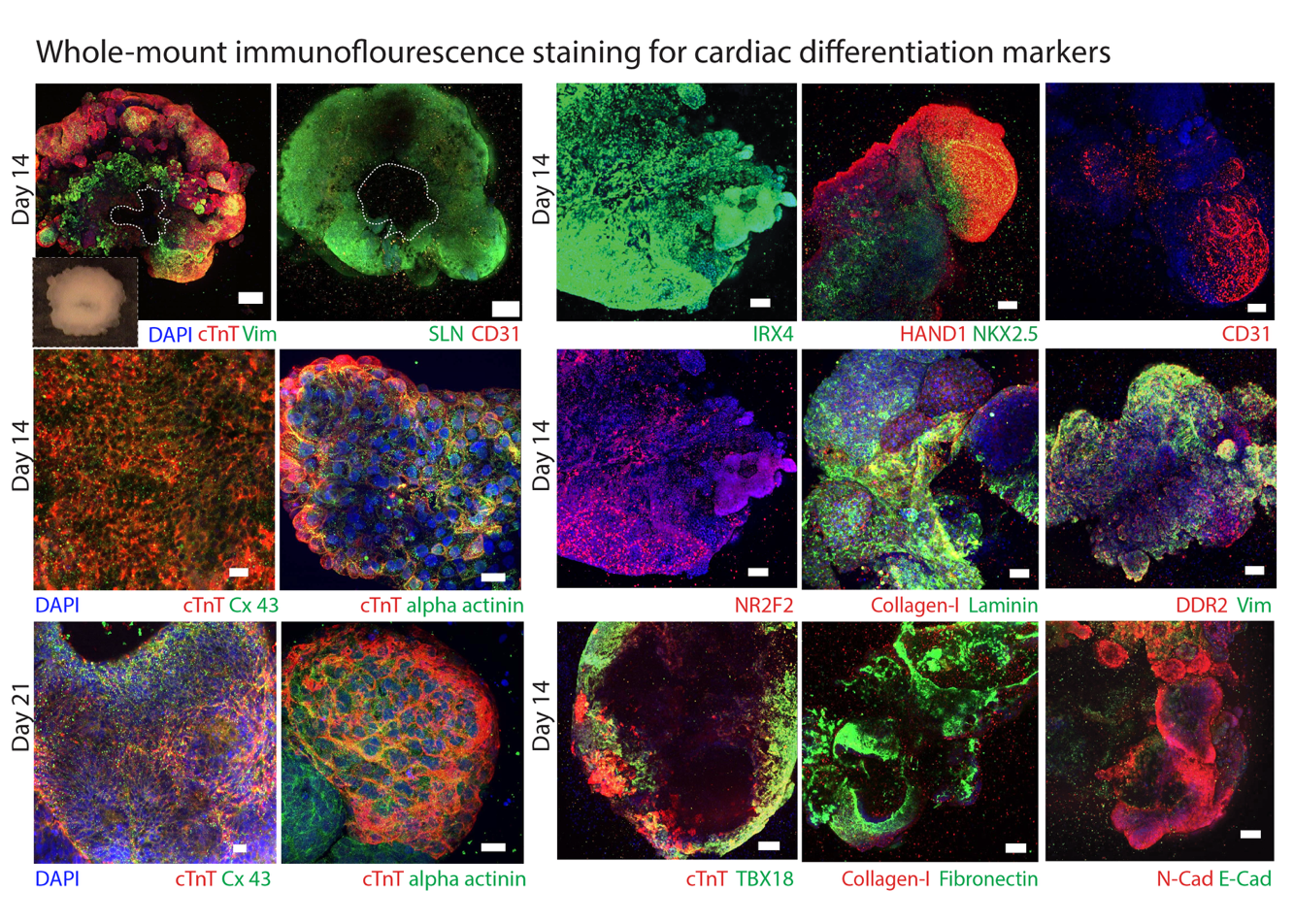


**Supplementary figure 6: Co-emergence of cardiomyocytes and multiple cardiac cell subtypes from iPSCs during in situ differentiation:** Immunofluorescence images of in situ differentiated bioprinted cardiac tissues confirming the emergence of cardiomyocytes and cardiac subtypes using a panel of antibodies on day 14 and day 21. The immunofluorescence analysis includes markers for cardiomyocytes (cTnT, connexin 43, sarcomeric alpha actinin), ventricular cardiomyocytes (IRX4), atrial cardiomyocytes (NR2F2, SLN), epicardial cells (TBX18), cardiac progenitors (HAND1, NKX2.5), fibroblasts (DDR2, vimentin), endothelial cells (CD31), EMT transition markers (E-cadherin, N-cadherin) and ECM secretion (collagen-I, fibronectin, laminin). The lumens of bioprinted ring constructs are indicated using a white dashed mark. (Scales are as follows: 200 μm for the images of the complete ring in b top left, 50 μm for collagen-laminin images, 20 μm for Cx43 and alpha actinin images, and 100 μm for all other images*)*

*
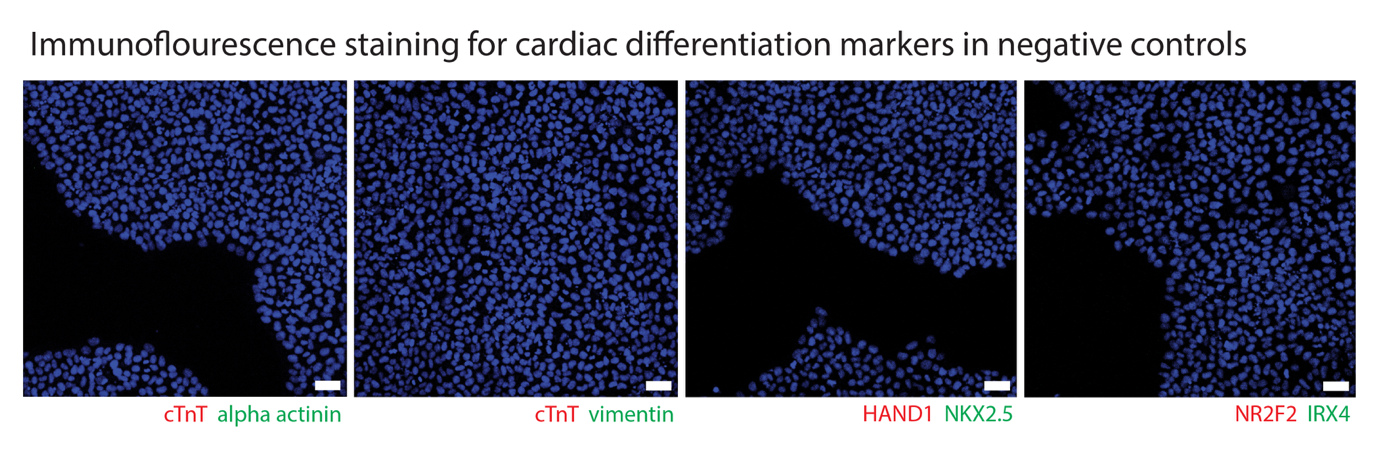
*

**Supplementary figure 7: Validation of cardiac differentiation marker specificity for immunofluorescence staining using negative control samples:** Immunofluorescence images of 2D iPSC cultures maintained in pluripotency media stained for cardiac differentiation markers. The negative staining validates the specificity of the markers used to evaluate the presence of cardiac progenitors (HAND1, NKX2.5, NR2F2, and IRX4), cardiomyocytes (cTnT), and cardiac fibroblasts (vimentin) in Figure 6. Scale bars are 50 μm.

Supporting Information S3 – Additional Table

**Table S3.1:** Cell sources

| Cell type | Catalogue no. | Company |
| --- | --- | --- |
| Gibco™ episomal hiPSC line | A18945 | Fisher Scientific |

**Table S3.2:** List of Materials and Reagents

| Material/Reagents | Catalog no. | Company |
| --- | --- | --- |
| PureCol®Collagen I: 3mg/ml | 5005 | Advanced Biomatrix |
| Invitrogen™ CellTracker™ Red CMTPX Dye | C34552 | Fisher Scientific |
| Corning™ Matrigel™ hESC-Qualified Matrix | 354277 | Fisher Scientific |
| mTeSR^TM^ Plus | 100-1130 | Stemcell Technologies |
| Gibco^TM^ Essential 8^TM^ medium | A2858501 | Fisher Scientific |
| Gibco™ DMEM/F-12, GlutaMAX™ supplement | 10565018 | Fisher Scientific |
| Gibco^TM^ RPMI 1640 medium |  | Fisher Scientific |
| Gibco™ TrypLE™ Select Enzyme 10X | A1217701 | Fisher Scientific |
| Accutase | 07920 or 07922 | Stemcell Technologies |
| Agarose, low EEO type I | A6013 | Merck |
| IWP 4 | 72554 | Stemcell Technologies |
| CHIR99021 | 72054 | Stemcell Technologies |
| Phalloidin–Tetramethylrhodamine B isothiocyanate | P-1951 | Merck |
| Gibco™ B27 minus insulin | A1895601 | Fisher Scientific |
| Gibco™ B27 supplement (50X), serum free | 17504044 | Fisher Scientific |
| Invitrogen^TM^ Calcein AM | C3100MP | Fisher scientific |
| Ethidium homodimer I solution | E1903 | Merck |
| Fluoroshield^TM^ with Dapi | F6057 | Merck |
| Isoproterenol hydrochloride | I6504 | Merck |
| Cal 520 | 21131 | AAT Bioquest |
| RT2 First Strand Kit | 330404 | Qiagen |
| RT2 SYBR Green ROX qPCR Mastermix | 330522 | Qiagen |
| Custom RT2 PCR Array 96-well plate | 330171 | Qiagen |
| µ-Dish 35 mm, high Glass Bottom | 81158 | ibidi |
| AggreWell™400 6-well Plate | 34425 | Stemcell Technologies |
| Anti-Adherence solution | 07010 | Stemcell Technologies |
| µ-plate 96 well | 89626 | ibidi |
| Antibodies | Catalog no. | Company |
| Collagen-I | Invitrogen MA126771 | Fisher Scientific |
| Vimentin | Invitrogen MA110459 | Fisher Scientific |
| CD31 Monoclonal Antibody (WM59) | Invitrogen MA126196 | Fisher Scientific |
| OCT 4 Polyclonal antibody | Invitrogen PA5-27438 | Fisher Scientific |
| SSEA-4 monoclonal antibody (MC-813-70) | Invitrogen 41-400 | Fisher Scientific |
| Cardiac Troponin T Monoclonal Antibody (13-11) | Invitrogen MA512960 | Fisher Scientific |
| Anti-Sarcomeric Alpha Actinin antibody | ab137346 | Abcam |
| SLN | 16848454 | Fisher Scientific |
| SOX2 | ab79351 | Abcam |
| Brachyury | 17769603 | Fisher Scientific |
| Ki67 | ab16667 | Abcam |
| Fibronectin | Invitrogen MA5-32509 | Fisher Scientific |
| Laminin | ab11575 | Abcam |
| HAND1 | Invitrogen MA5-25494 | Fisher Scientific |
| NKX 2.5 | Invitrogen 701622 | Fisher Scientific |
| IRX4 | Invitrogen PA5-97879 | Fisher Scientific |
| NR2F2 | ab41859 | Abcam |
| TBX18 | ab115262 | Abcam |
| DDR2 | ab63337 | Abcam |
| Connexin 43 | Invitrogen 71-0700 | Fisher Scientific |
| E-cadherin | ab40772 | Abcam |
| N-cadherin | 11571112 | Fisher Scientific |
| Fibronectin | Invitrogen MA532509 | Fisher Scientific |
| Goat- anti-mouse 594 | ab150116 | Abcam |
| Goat- anti-mouse 488 | ab150113 | Abcam |
| Goat- anti-rabbit 488 | ab150077 | Abcam |
| Goat-anti-mouse 647 | ab150115 | Abcam |

**Table S3.3:** Gene List for RT-PCR:

| Gene | Description code |
| --- | --- |
| OCT4- Pluripotent | Hs04260367_gH |
| SOX2- Pluripotent | Hs04234836_s1 |
| Nanog- Pluripotent | Hs02387400_g1 |
| OTX2- Ectoderm | Hs00222238_m1 |
| T- Mesoderm | Hs00610080_m1 |
| SOX17-Endoderm | Hs00751752_s1 |
| CDH1-E-cadherin | Hs01023895_m1 |
| CDH2-N-cadherin | Hs00983056_m1 |
| Ki67- Cell Proliferation | Hs01032443_m1 |
| GAPDH-Housekeeping | Hs99999905_m1 |

**Table S3.4:** Custom RT^2^ PCR array: Gene details

| GenBank | Symbol | Description |
| --- | --- | --- |
| NM_003380 | VIM | Vimentin |
| NM_006475 | POSTN | Periostin |
| NM_001613 | ACTA2 | Actin, Alpha 2, smooth muscle, aorta |
| NM_006182 | DDR2 | Discoidin domain receptor tyrosine kinase 2 |
| NM_004387 | NKX2-5 | NK2 homeobox 5 |
| NM_002052 | GATA4 | GATA binding protein 4 |
| NM_003281 | TNNI1 | Troponin I type 1 (skeletal, slow) |
| NM_000364 | TNNT2 | Troponin T type 2 (cardiac) |
| NM_000363 | TNNI3 | Troponin I type 3 (cardiac) |
| NM_000165 | GJA1 | Gap junction protein, alpha 1, 43kDa |
| NM_181703 | GJA5 | Gap junction protein, alpha 5, 40kDa |
| NM_000891 | KCNJ2 | Potassium inwardly-rectifying channel, subfamily J, member 2 |
| NM_000719 | CACNA1C | Calcium channel, voltage-dependent, L type, alpha 1C subunit |
| NM_000335 | SCN5A | Sodium channel, voltage-gated, type V, alpha subunit |
| NM_001681 | ATP2A2 | ATPase, Ca ++ transporting, cardiac muscle, slow twitch 2 |
| NM_021223 | MYL7 | Myosin, light chain 7, regulatory |
| NM_000432 | MYL2 | Myosin, light chain 2, regulatory, cardiac, slow |
| NM_000257 | MYH7 | Myosin, heavy chain 7, cardiac muscle, beta |
| NM_002471 | MYH6 | Myosin, heavy chain 6, cardiac muscle, alpha |
| NM_002046 | GAPDH | Glyceraldehyde-3-phosphate dehydrogenase |
